# Psychometric properties of hierarchical psychiatric symptoms on the general population

**DOI:** 10.1371/journal.pmen.0000633

**Published:** 2026-06-17

**Authors:** Takafumi Soda, Shingo Murata, Asako Toyama, Shinsuke Suzuki, Yoshihiko Kunisato, Kentaro Katahira, Yuichi Yamashita

**Affiliations:** 1 Department of Information Medicine, National Institute of Neuroscience, National Center of Neurology and Psychiatry, Kodaira, Tokyo, Japan; 2 Department of Electronics and Electrical Engineering, Faculty of Science and Technology, Keio University, Minato, Japan; 3 Graduate School of Social Data Science, Hitotsubashi University, Kunitachi, Tokyo, Japan; 4 Graduate School of the Humanities, Senshu University, Kawasaki, Kanagawa, Japan; 5 Centre for Brain, Mind and Markets, Faculty of Business and Economics, The University of Melbourne, Melbourne, Australia; 6 Faculty of Social Data Science, Hitotsubashi University, Kunitachi, Tokyo, Japan; 7 HIAS Brain Research Center, Hitotsubashi University, Kunitachi, Tokyo, Japan; 8 Human Informatics and Interaction Research Institute, National Institute of Advanced Industrial Science and Technology (AIST), Tsukuba, Japan; 9 Department of Cognitive and Psychological Sciences, Graduate School of Informatics, Nagoya University, Nagoya, Japan; National University of Singapore, SINGAPORE

## Abstract

Although psychiatric disorders have conventionally been treated categorically, recent research indicates a continuous and hierarchical structure among psychiatric symptoms, with a general psychopathology factor (*p*-factor) at the top and several specific factors at lower levels. However, the understanding of the psychometric properties of these factors in the general population remains limited. In this study, by analyzing responses from approximately 1000 non-Western individuals across three datasets to various diagnostic questionnaires, we extracted general and specific factors using diverse hierarchical structures and factor numbers. Comparative analysis revealed distinct properties of the general and specific factors, regardless of model assumptions. Specifically, the general factor explained a greater proportion of shared variance in the data and exhibited higher internal consistency than the specific factors. Consistent with the concept of the *p*-factor, the general factor was associated with a history of diagnoses across various diagnostic categories. Conversely, the content of the specific factors varied depending on the factor numbers, and some corresponded to traditional diagnostic categories, indicating that specific factors may be advantageous for describing individual differences. Our findings, we believe, provide a scaffold for disentangling the complex structure among psychiatric symptoms.

## Introduction

Categorical diagnostic approaches, such as the Diagnostic and Statistical Manual of Mental Disorders (DSM), have contributed common languages to psychiatric research and clinical practice [1]. Although their advantages are significant, several limitations associated with the categorical approach have been highlighted. For instance, it is common for individuals to be diagnosed with conditions that span multiple categories simultaneously [2]. The situation is further complicated by the observation that similar symptoms may appear across different diagnostic categories (transdiagnostic symptoms). This complexity in categorical approaches is exacerbated by the criteria for the same diagnosis encompassing different combinations of symptoms, leading to notable heterogeneity in classifications [3]. This heterogeneity is indicative of a low correspondence between diagnostic categories and biological features, which limits the efficacy of diagnoses in predicting prognosis and treatment responses [4]. Consequently, efforts are ongoing to develop a more precise diagnostic system for mental disorders.

The dimensional approach has been recognized as an alternative to conventional categorical diagnostics [4,5]. This approach attempts to profile individuals using combinations of continuous psychiatric dimensions, regardless of their diagnostic history. The dimensional approach assumes no clear boundaries between health and pathology [6]. Empirical studies employing factor analysis have identified common latent transdiagnostic dimensions (e.g., internalizing and externalizing dimensions) among similar psychiatric symptoms [5,7]. Additionally, recent theoretical frameworks acknowledge that transdiagnostic dimensions are organized hierarchically. For example, the Hierarchical Taxonomy of Psychopathology (HiTOP) consortium assumes that individual psychiatric symptoms fall into one of several latent dimensions (i.e., internalizing, thought disorder, detachment, disinhibited, and antagonistic externalizing) [5,8]. Moreover, these dimensions are also correlated, giving rise to a higher-order dimension termed the general psychopathology factor [9,10]. The general psychopathology factor, also known as the “*p*-factor,” has been useful in predicting mental health outcomes and cognitive performance and can be utilized in research and clinical practice [11].

Alongside theoretical advancements in the dimensional approach, research designs investigating the content and properties of psychiatric dimensions have been further refined [12,13]. Studies based on the continuum hypothesis in general populations have increased, largely due to advancements in online methods that enable large-scale sample testing [13,14]. This approach has contributed to elucidating latent dimensions of psychiatric symptoms within the general population, in contrast to earlier research focusing on specific groups, such as case-control studies. Recent studies have combined questionnaires from different diagnostic categories for mental disorders and conducted factor analyses on the pooled items [13]. This transdiagnostic approach at the item level (item-level joint factor analysis) may contribute to the reconstruction of mental disorder dimensions in a bottom-up manner. Item-level joint factor analyses have revealed that non-categorical and transdiagnostic latent factors can be robustly extracted [13–18]. Furthermore, these studies have reported on the utility of these factors, specifically that they are more strongly and clearly associated with external criteria, such as deficits in goal-directed control [13,14,17].

However, research on psychiatric symptom dimensions using item-level joint factor analyses in general populations remains immature. Specifically, inadequate exploration of different types of *hierarchical* structures and varying *numbers* of factors has led to the psychometric properties (i.e., reliability and validity) of these dimensions to be poorly understood in the general population.

**Fig 1:**
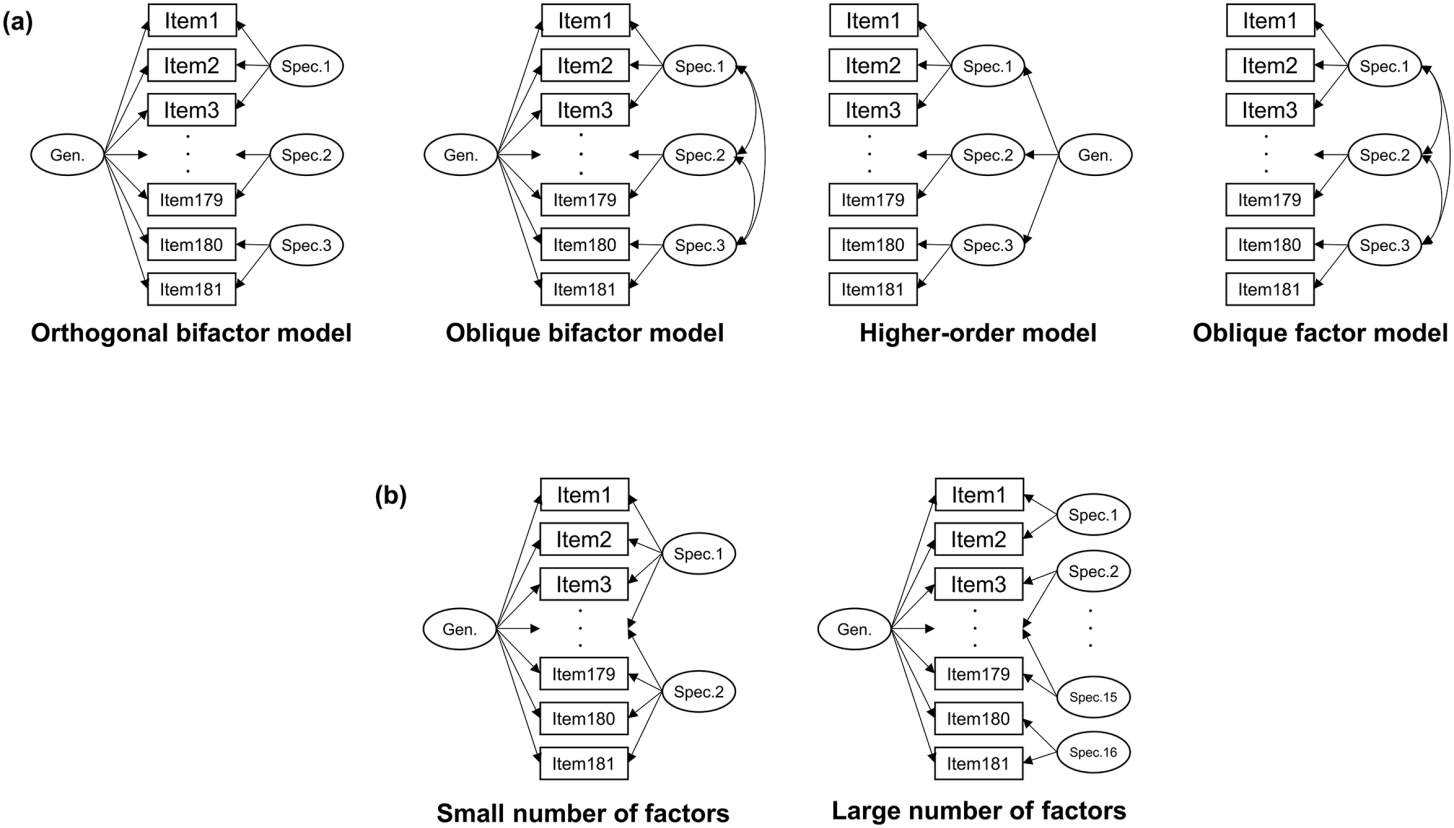
Schematic representation of the analyzed factor models. The reliability and validity of the general and specific factors were compared under various model assumptions, specifically (a) types of hierarchical structure and (b) numbers of factors. The compared factor models have different hierarchical structures: orthogonal bifactor, oblique bifactor, and higher-order factor models **(a)**. The bifactor model entails a hierarchical factor structure wherein a general factor directly influences all observed items. The oblique bifactor model allows correlations between specific factors, whereas the orthogonal bifactor model does not. The higher-order factor model involves the general factor loading onto all specific factors. A non-hierarchical model, the oblique factor model, was also included in the comparison. In addition to examining the structures of the factor models, the number of factors was also compared **(b)**. For instance, in Dataset-1, which comprised 181 items, the comparison encompassed factor models ranging from three to 17 factors. Spec.: specific factor; Gen.: general factor.

The first challenge is that psychometric properties of the psychiatric dimensions in the general population have not been sufficiently investigated under the assumption of a hierarchical structure [19–21]. Despite the recognized importance of hierarchy in psychiatric symptoms, many item-level joint factor analyses have not incorporated this assumption. For instance, several studies have relied solely on oblique factor models (in this paper, factor models that do not include higher-order factors are referred to as oblique factor models), which overlook hierarchical structures [13,14,16–19]. Although one exceptional study assumed a hierarchy, it focused only on internalizing symptoms and examined restricted types of hierarchical structures [15]. Furthermore, a debate persists over the appropriate hierarchical structure for psychiatric dimensions [22–24]. For example, previous research has pointed out that while bifactor models tend to show superior model fit, higher-order models offer greater interpretability of latent factors [24,25]. Here, the bifactor model posits that a general factor directly influences all observed items, whereas the higher-order factor model assumes that a general factor influences specific factors, which in turn influence the observed items (Fig 1a). Moreover, when assuming a hierarchical structure of psychiatric symptoms, it is plausible that correlations may arise among specific factors as their number increases. Nevertheless, prior studies have primarily employed orthogonal bifactor models, and the advantages of oblique bifactor models have not been thoroughly investigated. This limited exploration of hierarchy in factor structures has led to an inadequate understanding of the psychometric properties of hierarchical psychiatric dimensions.

Further complications arise because research on the psychometric properties of hierarchical factors often focuses only on a small subset of factors [26–30]. For example, Polek et al. (2018) [27] determined only four factors from 116 items as transdiagnostic psychopathological traits (maladaptive personality), which may not adequately reflect the properties of the questionnaires, given that standard questionnaires construct three to five factors based on 20–30 items (e.g., subscales contain 6.54 items on average [21]). Therefore, it is necessary to construct transdiagnostic factors from the bottom up to ensure that an appropriate number of factors is considered [12,31]. A larger number of factors may provide richer individual descriptors and increase the clinical applicability of the hierarchical hypothesis.

Investigating various parameters, such as hierarchical structures and the number of factors, may better clarify their psychometric properties in the general population. For instance, examining a large number of factors can provide insight into these poorly understood psychometric properties. Additionally, the distinctions between general and specific factors may be elucidated by investigating their consistent properties independent of hierarchical structures and factor numbers. Understanding these parameters can shed light on how these factors fluctuate based on different configurations, offering valuable guidance for future hierarchical factor analyses.

This study aimed to examine the psychometric properties of general and specific factors estimated from a wide range of questionnaires related to psychiatric symptoms. In particular, we investigated the reliability and validity of hierarchical psychiatric symptoms using exploratory factor analysis (EFA). The hierarchical structures we examined included orthogonal bifactor, oblique bifactor, and higher-order factor models (Fig 1a). This choice was influenced by ongoing debates about whether higher-order or bifactor models better represent the data, as well as by the relatively limited examination of oblique bifactor models [22–24]. Furthermore, when comparing models that include a general factor—such as bifactor and higher-order models—it is useful to employ a multifactor model without a general factor as a baseline. This allows for a clearer evaluation of the added value of incorporating a general factor. Additionally, the numbers of factors were exhaustively compared (Fig 1b). We attempted to cover factor models with a larger number of specific factors that have not been investigated in previous studies. These candidates were carefully evaluated based on comparisons of their psychometric properties, including dimensionality, internal consistency (reliability), convergent and discriminant validity, and interpretability. Furthermore, we visualized the multilayered hierarchy of psychiatric symptoms [12,32]. Finally, the findings were validated through replication using multiple independently collected datasets.

## Materials and methods

### Ethics statement

The datasets used in this study were obtained from the original data collectors with appropriate permission for secondary analysis. All data were originally collected under ethical protocols approved by the respective institutions. For details of the ethical procedures, please refer to the original publications for each dataset. In all the datasets, participants provided informed consent by clicking “I agree” after reading information about the survey. None of the collected or received data included any information that could identify individual participants. All procedures were carried out in accordance with the provisions of the World Medical Association Declaration of Helsinki.

### Datasets

Three datasets [17,18,33] that included a wide range of questionnaires measuring psychiatric symptoms were analyzed. All datasets were collected independently for different purposes using online surveys in Japan.

The first dataset (Dataset-1) was obtained to analyze the relationship between transdiagnostic symptoms and drawing behavior [18]. It included responses to questionnaires and diagnostic history (schizophrenia, mood disorders, anxiety disorders, obsessive-compulsive disorders, developmental disorders, and unspecified disorders) from 1163 (female = 690; male = 473) participants with a mean age of 38.06 years (standard deviation (SD) = 10.29). The following questionnaires were administered: the Schizotypal Personality Questionnaire-Brief (SPQB) for evaluating schizotypal personality disorder symptoms, comprising 22 items with binary responses [34]; the Obsessive-Compulsive Inventory (OCI) for evaluating obsessive-compulsive symptoms, comprising 42 items with a five-point scale [35]; the Patient Health Questionnaire (PHQ) for evaluating depression symptoms, comprising nine items with a four-point scale [36]; the State-Trait Anxiety Inventory (STAI) for evaluating anxiety symptoms, comprising 40 items with a four-point scale [37]; the Autism-Spectrum Quotient (AQ) for evaluating autism spectrum disorder symptoms, comprising 50 items with binary responses [38]; and the Adult Attention-Deficit Hyperactivity Disorder (ADHD) Self-Report Scale version 1.1 (ASRS) for evaluating ADHD symptoms, comprising 18 items with a five-point scale [39]. This study primarily reports the results of Dataset-1 [18]; Datasets-2 and -3 were used for the validation study.

The second dataset (Dataset-2) was collected to investigate the reliability and validity of the DSM-5 Level 1 Cross-Cutting Symptom Measure [33]. This dataset included responses from 985 participants (female = 607; male = 378) with a mean age of 38.87 years (SD = 9.91). In addition, their diagnoses were obtained (the diagnostic categories were the same as those in Dataset-1). This dataset covered a wider range of symptoms than the other two: SPQB, PHQ, OCI, the Insomnia Severity Index (ISI) for evaluating insomnia, comprising seven items with five-level responses [40]; the Alcohol Use Disorders Identification Test (AUDIT) for evaluating use disorders, comprising eight items with a five-point scale and two items with a three-point scale [41]; state anger from the State-Trait Anger Expression Inventory (STAXI) for evaluating irritability symptoms, comprising 10 items with four-level responses [42]; the Dissociative Experiences Scale-II (DES) for evaluating dissociative symptoms, comprising 28 items with an 11-point scale [43]; and the Millon Clinical Multiaxial Inventory-II Borderline Scale (MCMI) for evaluating borderline personality traits, comprising 17 items with binary responses [44].

The third dataset (Dataset-3) was collected to investigate the relationship between transdiagnostic symptoms and computational phenotypes in decision-making tasks [17]. This study did not include participants diagnosed with mental disorders; 1,900 individuals participated (mean age = 47.77 years; SD = 11.62). Two separate groups answered the same questionnaires but performed different decision-making tasks (i.e., reward-seeking [male = 445 and female = 494] and loss-avoidance [male = 455 and female = 506] tasks). The following questionnaires were administered: SPQB, OCI, STAI, the Self-Rating Depression Scale (SDS) for evaluating depression symptoms, comprising 20 items with four-level responses [45]; and the Barratt Impulsivity Scale (BIS) for evaluating attention-deficit/hyperactivity symptoms, comprising 30 items with five-level responses [46].

Participants were limited to those who could read Japanese. Detailed demographic data are presented in S1 Text. All questionnaires administered in these studies were translated into Japanese and confirmed to have sufficient reliability and validity. Scores were transformed such that higher scores indicated greater symptom severity.

The datasets used in this study were obtained from the original data collectors with appropriate permissions for secondary analysis. Dataset-1 and Dataset-3 were received in September 2020, and Dataset-2 was received in March 2021. All data were originally collected under ethical protocols approved by the respective institutions. For details of the ethical procedures, please refer to the original publications for each dataset [17,18,33]. Across all datasets, participants provided informed consent by clicking “I agree” after reading information about the survey. None of the collected or received data included any information that could identify individual participants. All procedures were carried out in accordance with the provisions of the World Medical Association Declaration of Helsinki.

### Model comparisons

The reliability and validity of the factors identified based on the four types of model structures were compared: orthogonal bifactor, oblique bifactor, higher-order factor, and oblique factor models (Fig 1a). Bifactor and higher-order factor models were used because they represent hierarchical factor models but remain highly controversial [22–24]. The oblique bifactor model allows for correlations between specific factors, whereas the orthogonal bifactor model does not. The oblique factor model was used as a baseline. Additionally, we investigated the effects of the number of factors (Fig 1b) because we could not determine a clear number of factors using conventional methods, as described in the “Results” section.

Confirmatory factor analysis (CFA) is useful for comparing models using fit indices and information criteria, and for testing whether hierarchical models fit better than non-hierarchical ones. However, CFA requires researchers to predefine which items load on which factors. In our case, each dataset included over 100 items from diverse questionnaires, making it difficult to apply a consistent rule for factor assignments across all datasets. Due to this complexity, we did not conduct CFA in this study; instead, all factor models were estimated using exploratory factor analysis (EFA) with maximum likelihood estimation based on polychoric correlation matrices. Polychoric correlations were used because the item scores were ordinal. Accordingly, all scores were treated as ordinal variables, and no additional procedures to homogenize the response formats (e.g., rescaling) were performed. Specifically, the correlation matrix was computed using the *hetcor()* function from the *polycor* package. Orthogonal bifactor, oblique bifactor, and oblique factor models were constructed using the bifactor, biquartimin, and promax rotations, respectively. To align an equal number of general and specific factors among all factor models, the higher-order factor model with N factors (one general and N−1 specific factors) was estimated by applying the Schmid-Leiman transformation to the oblique factor model with N−1 factors using the *omega()* function with promax rotation in the R package *psych*. To ensure convergence across all conditions during the EFA, the maximum number of iterations for the rotation algorithm was increased from the default threshold of 1000–50000.

### Metrics

#### Dimensionality.

To examine the validity of the factor structure, it is important to determine the number of latent factors into which the observed variables can be summarized. Explained common variance (ECV) is primarily used as an indicator of unidimensional/multidimensional structures when assuming a hierarchical factor structure [21]. ECV is the ratio of variance explained by the general factor to that explained by all common factors. Therefore, ECV ranges from zero to one, with higher values reflecting stronger effects of the general factor. Conventionally, if ECV values are higher than  .7, the common variance is considered to be explained by an “essentially unidimensional” factor [19,21]. ECVs for specific factors were also investigated for comparison.

We also conducted a principal component analysis (PCA) to visually inspect the scree plot and assess the proportion of variance explained by the first principal component. Unlike factor analysis, which depends on the number of factors and the rotation method used, PCA allows for the examination of the pure variance explained by the first component.

Furthermore, conventional methods to estimate the number of factors, such as the minimum average partial (MAP) criterion, parallel analysis, the Kaiser-Guttman criterion, and scree plots, were also performed using the R package *psych*. However, as discussed in the “Results” section, these standard estimation methods were unstable. As an alternative approach, we considered using conventional fit indices (e.g., TLI: the Tucker–Lewis Index) to compare models in our analysis; however, we chose not to rely on them for the following reasons. First, in a saturated model where all paths are freely estimated, as in EFA, models with the same number of factors yield identical fit statistics regardless of whether the structure is an oblique (non-hierarchical) or a bifactor (hierarchical) model. Second, although we investigated several fit indices (see Table C in S1 Text), their values tended to increase as the number of factors grew. These solutions often resulted in factors that were difficult to interpret and did not represent meaningful dimensions of psychiatric symptoms. Additionally, the results of standard methods for determining the number of factors, including fit indices, were inconsistent. Therefore, rather than relying solely on fit indices, we conducted a systematic exploratory analysis across an extensive range of factor solutions to evaluate the models from diverse perspectives. Such an approach enables the identification of stable and clinically meaningful dimensions of psychopathology.

#### Internal consistency.

Internal consistency, a measure of reliability, determines whether the items comprising a scale for a psychological construct (e.g., depression, anxiety) truly measure a single construct. As indices of internal consistency, two types of omega coefficients were used, namely omega reliability $\omega_{\text{t}}$ and omega hierarchical reliability $\omega_{\text{h}}$ [20,21]. Omega coefficients, similar to Cronbach’s α coefficient, are indices of internal consistency. Omega coefficients are model-based estimates of reliability and are weighted using the factor structure. Conversely, Cronbach’s α is calculated only from the observed variance-covariance matrix and does not assume a particular factor structure, such as equal loadings [19].

Both $\omega_{\text{t}}$ and $\omega_{\text{h}}$ are ratios of variance explained by factors to the variance of the total score. While $\omega_{\text{t}}$ focuses on all factors in the overall model, $\omega_{\text{h}}$ focuses on a single factor, not necessarily limited to the general and specific factors. The comparison between $\omega_{\text{t}}$ and $\omega_{\text{h}}$ allows for the analysis of whether the variance is due to general or specific factors [20]. When $\omega_{\text{h}}$ is high (>.80), the majority of variance is attributable to a single common source [21]. In addition, a high $\omega_{\text{h}}$ is needed to warrant psychometric interpretation, although the cut-off point is disputed [19]. These indices are usually used in CFA but can also be computed for exploratory models [20].

### Association strength: Label-independent analysis

We expected the general factor to exhibit stable and strong associations with various diagnoses because it is conceptualized as a common factor across all mental disorders. Conversely, specific factors were expected to correlate with only a limited number of diagnoses, as they are conceptualized as being specific to particular diagnoses. Therefore, specific factors are expected to display both strong and weak correlations with various diagnoses and to demonstrate weaker associations when averaged across diagnoses. To confirm this hypothesis, association strength was exhaustively investigated for all factor models. The compared models were identical in dimensionality and internal consistency. The procedure to quantify association strength was as follows: in the studies for Datasets-1 and -2, participants were asked whether they had been diagnosed with any of the aforementioned mental disorders. Therefore, the polyserial (biserial) correlations between diagnoses and factor scores could be quantified. The averages of the polyserial correlations across the diagnoses for each factor score were used to assess the association strength of the respective factors. For example, the association strength of the general factor was determined by calculating the average polyserial correlation between the factor score of the general factor and the diagnoses.

Factor scores were estimated using the ten Berge method. For this estimation, in addition to the parameters derived from the factor analysis, we utilized Pearson correlations of the observed data for the inter-item correlations, rather than polychoric correlations. This approach was chosen because it improved the recovery of factor correlations in the oblique bifactor model and stabilized the association with the diagnosis in the higher-order factor model. As indices of the strength of association, polyserial correlations were used because diagnoses were obtained using binary responses (i.e., whether the disorder had been experienced), while the factor scores were continuous. Factor scores, rather than structural equation modeling (SEM), were used to analyze association strength. Indeed, estimation errors are smaller with SEM than with factor scores. However, EFA and factor scores were used instead of SEM because model construction and convergence are difficult with numerous observed variables (number of questionnaire items). Moreover, in the higher-order factor model (Schmid-Leiman transformation), the solutions in SEM and multiple regression analysis are not correctly estimated when both general and specific factors are simultaneously included as independent variables, because the general factor is a transformation of the specific factors in this model. Consequently, the correlations between the general and specific factors become very high, leading to multicollinearity [22]. Therefore, when comparing multiple hierarchical models, correlation analysis is more appropriate than multiple regression.

### Interpretability

Although the statistical properties of the estimated factors were sufficient, the factors would be meaningless if they were inconsistent with theoretical hypotheses and clinical intuition. Therefore, interpreting the extracted general and specific factors is important. We selected the factor solution with as many factors as possible to provide representative results for interpretability, because previous studies have focused on fewer factors, even though a wide variety of specific factors may be beneficial. We investigated whether the estimated specific factors of the oblique bifactor models (the reason for selecting the oblique bifactor model is reported in the “Results” section) were interpretable, starting from solutions with a large number of factors. We set the necessary condition for interpretation as the presence of at least four items with loadings greater than 0.30. We then examined whether the factors satisfying these conditions constituted a single construct that could be labeled. Specific factors were labeled based on items with factor loadings of 0.30 or greater. While considering the overall content of these items, we prioritized the characteristics of items with the highest factor loadings. In this process, the first author initially proposed the labels based on the item content, which were then critically reviewed by the final author. These labels are intended only for the convenience of presenting our results clearly, and we do not intend for them to be reused or generalized in the future.

### Convergent and discriminant validity: label-dependent analysis

Convergent and discriminant validity were assessed by verifying whether the obtained factors were associated with the intended constructs and not with unintended ones. For this purpose, we performed multiple logistic regression analyses for the diagnoses using the obtained labeled factors. Although we again employed association strength, this label-dependent analysis allowed us to examine convergent and discriminant validity in greater detail by analyzing the relationships between the interpreted specific factors and diagnostic categories. We hypothesized that the general factor would be associated with diagnoses across all diagnostic categories, whereas each specific factor would be correlated with only one diagnosis; for example, a specific factor related to depression would be correlated with a diagnosis of mood disorders.

### Multi-layered hierarchy of psychiatric symptoms

We analyzed and visualized the overall hierarchical structure of psychiatric symptoms by integrating factor models across multiple levels, from fewer to several factors [12,32]. First, factor scores were calculated for the factor models using EFA with biquartimin rotation (oblique bifactor model). Second, the panels with label names were plotted such that the height of each panel represented the ECV of the respective factor. Third, correlation coefficients were calculated between the factor scores of models with consecutive numbers of factors, such as models with five and six factors. Because the factor scores showed strong non-normality (Table D in S1 Text), we calculated the correlations between them using Spearman’s method. The thickness of the curves between these labels corresponded to the absolute values of the correlation coefficients of the factor scores (a softmax function was used to highlight the size of the correlation coefficients for better visibility). The specific factor labels were determined following the same procedure described in the “Interpretability” section. Additionally, specific factors at both higher and lower hierarchical levels were considered.

### Software

All analyses were performed using R software. The R packages *polycor*, *psych*, *lavaan*, and *BifactorIndicesCalculator* were used to estimate polychoric correlations, EFA, CFA, ECV, and $\omega_{\text{t}}$ reliabilities.

## Results

### Dimensionality

The correlation analysis showed that items not only within the same questionnaire but also across different questionnaires were moderately correlated in Dataset-1. For example, the average correlation coefficient between pairs of items across all questionnaires was  .221 (standard deviation = .144). Furthermore, the first principal component explained 24.7 percent of the total variance, followed by 7.37 and 4.52 percent.

Standard factor analysis methods, including the MAP criterion, parallel analysis, the Kaiser-Guttman criterion, and scree plots, were applied to investigate the number of factors. However, because of the inconsistent results from these methods, the number of factors could not be accurately specified. For example, scree plot analysis based on the decay of eigenvalues suggested a one-factor solution (Fig A in S1 Text). On the other hand, the Kaiser-Guttman criterion, using the number of eigenvalues over 1.0, suggested 34 factors, and the BIC achieved a minimum with 37. The MAP criterion suggested 17 factors, and parallel analysis suggested 23.

Because the suggested factor numbers were inconsistent, we exhaustively analyzed and compared the four types of model structures and various numbers of factors in the following analyses. A three-factor solution (one general and two specific factors) was selected as the minimum number. This number was chosen because it represents the minimum requirement for higher-order factor models. To determine a model wherein all factors are interpretable, it was necessary to explore solutions that incorporate a sufficiently large number of factors. Preliminary analyses indicated that including more than 17 factors, as suggested by the MAP criterion, resulted in uninterpretable solutions, such as instances wherein a factor was represented by only a single item with a high factor loading. Therefore, to ensure the interpretability of all factors, subsequent analyses were conducted using models with a factor range of 3–17.

**Fig 2:**
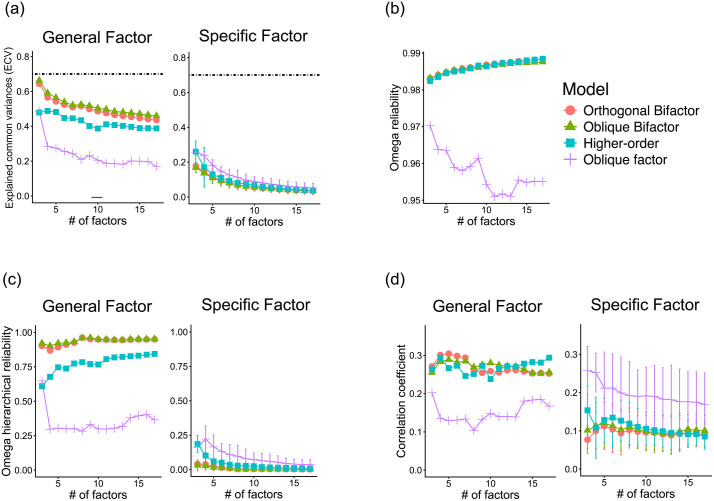
Comparisons of the psychometric properties. **(a)** Explained common variances (ECVs) of the general and specific factors in each factor model. ECV is derived from the proportion of variance explained by each factor and serves as an indicator of dimensionality. If ECV values are higher than  .7 for the general factor (dot-dash- line in the figure), the common variance is explained by an “essentially unidimensional” factor [19,21]. **(b)** Omega reliabilities $\omega_{\text{t}}$ in each factor model. **(c)** Omega hierarchical reliabilities$\omega_{\text{h}}$ for general and specific factors in each factor model. **(d)** Association strengths of general and specific factors in each factor model with diagnoses of mental disorders. The strength of association was determined by averaging the polyserial correlations. The first factor in the oblique factor model was used as the general factor for comparison. The number of factors (on the x-axis) includes both the general and specific factors. Error bars represent the standard deviations among multiple specific factors.

Fig 2a illustrates the ECVs of the general and specific factors as the model structures and factor numbers were varied. The ECV of the general factor in the bifactor structure approached the threshold (.7) when the number of factors was small, although it did not exceed it. Moreover, the ECV of the general factor decreased as the number of factors increased but remained above  .4 even with a large number of factors. The ECVs of the general factors in the higher-order factor model were lower than those in the oblique and orthogonal bifactor structures. The ECVs of specific factors in the hierarchical models and the first factor in the oblique solutions were below 30 percent. These ECV results suggest that the variance was mainly explained by the general factor, although there was insufficient evidence to strongly support a unidimensional structure; therefore some specific latent factors might also exist.

### Internal consistency

Reliability was examined using omega coefficients, which represent internal consistency when assuming that the factor model has a hierarchy. Fig 2b represents omega reliability $\omega_{\text{t}}$ when model structures and factor numbers were changed. The values were very high for all hierarchical structures and factor numbers, suggesting that the total scores of these scales had sufficient internal consistency. Although the oblique factor model did not have a hierarchical structure, omega reliability $\omega_{\text{t}}$ was calculated using the first factor as the general factor for comparison. Therefore, although omega reliabilities $\omega_{\text{t}}$ were over  .95 in the oblique factor model, these values were lower than those of the hierarchical models.

To investigate the relative influences of general and specific factors on reliability, their omega hierarchical reliability $\omega_{\text{h}}$ was analyzed (Fig 2c). The $\omega_{\text{h}}$ of the general factor in orthogonal and oblique bifactor models showed very high values (approximately  .90) regardless of the number of factors. The $\omega_{\text{h}}$ of the general factor in the higher-order factor model was slightly lower than that in the orthogonal and oblique bifactor models, although it improved as the number of factors increased (Fig 2c, left panel). Similar to $\omega_{\text{t}}$, $\omega_{\text{h}}$ was calculated in the oblique factor model using the first factor as the general factor. The reliability of the first factor was extremely low in the oblique factor model with more than four factors. Furthermore, most of the $\omega_{\text{h}}$ values for the specific factors were below  .2 regardless of the model structure. Additionally, $\omega_{\text{h}}$ tended to decrease as the number of factors increased. These results suggest that the internal consistency of the composite scores for each factor predominantly depended on the general rather than the specific factors, indicating that the specific factors may lack sufficient reliability.

### Association strength: Label-independent analysis

Association strength for the diagnostic categories was analyzed using factor scores. Fig 2d shows the results of the association strength across model structures and factor numbers. In the hierarchical models, association strength ranged roughly from  .25 to  .30. There was no clear relationship between the number of factors and association strength for the general factor. Conversely, the specific factors exhibited lower association strength (approximately  .10) compared to the general factor. Additionally, there was substantial variance in the association strength of the specific factors, indicating that they may demonstrate relatively high associations in certain cases (Fig 2d, right). The first factor of the oblique factor model showed lower association strength than the hierarchical models (Fig 2d, left). The oblique factor model sometimes demonstrated second and subsequent factors with greater association strength than its first factor (Fig 2d).

### Interpretability

The oblique bifactor (biquartimin) solution was selected as the target model for interpretation. This is because it is natural for multiple specific factors in the lower-order layer to exhibit correlations with each other when the number of factors is large, although the ECV, internal consistency, and association strength of the hierarchical models are comparable. Interpretations were attempted starting from the 17-factor solution. Some factors could not be clearly interpreted as a single construct in the solutions with 13–17 factors. Specifically, these factors had relatively low factor loadings and related to too few items (a criterion of fewer than four items exceeding 0.30 was applied). In other cases, the factor with the lowest variance explanation ratio had factor loadings simultaneously from items that were semantically unrelated.

**Fig 3:**
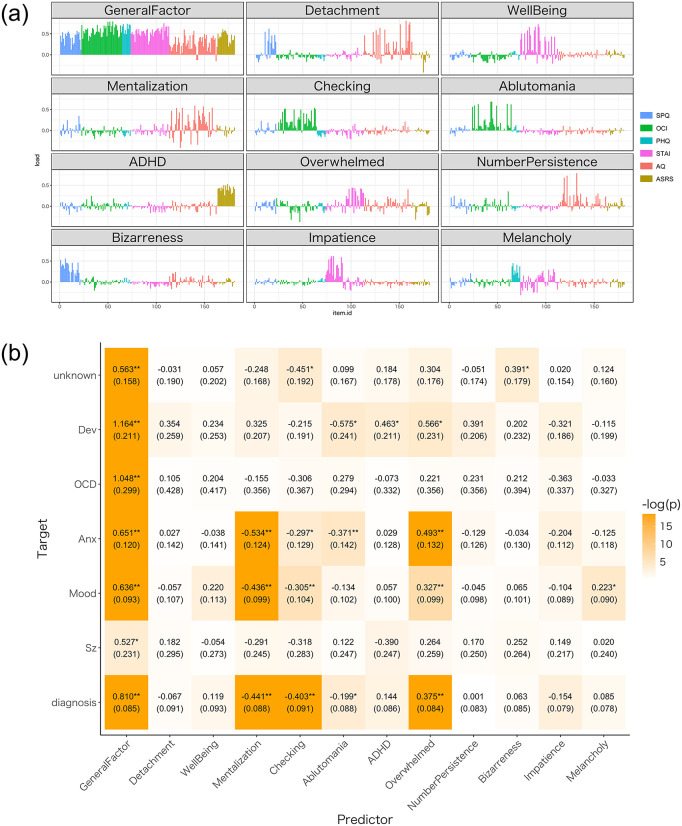
Results of the oblique bifactor model with 12 factors. **(a)** Factor loadings. The horizontal axis indicates the items constituting the questionnaire. The vertical axis represents factor loadings. AQ: the Autism-Spectrum Quotient; ASRS: the Adult Attention-Deficit Hyperactivity Disorder Self-Report Scale; OCI: the Obsessive-Compulsive Inventory; PHQ: the Patient Health Questionnaire; SPQ: the Schizotypal Personality Questionnaire-Brief; STAI: the State-Trait Anxiety Inventory. **(b)** Multiple regression analysis of general and specific factors for diagnoses of psychiatric disorders to assess convergent and discriminant validity. Values in each cell represent regression coefficients and standard deviations. The color scale reflects the p-values, with darker colors indicating smaller p-values. Specifically, the p-values were added to a small constant (10^-8^) and then transformed into negative natural logarithms for visualization. Dev: Developmental disorders; OCD: Obsessive-compulsive disorders; Anx: Anxiety disorders; Mood: Mood disorders; Sz: Schizophrenia; diagnosis: diagnoses of any mental disorders. ** p<0.01; * p<0.05.

All factors in the solution with 12 factors (one general and 11 specific factors) were interpretable (Fig 3a). The items with the highest loading on the general factor were “I frequently get nasty thoughts and have difficulty getting rid of them” (item 33 in OCI; β = 0.792), “I am upset by unpleasant thoughts that come into my mind against my will” (item 30 in OCI; β = 0.790), and “I find it difficult to control my thoughts” (item 28 in OCI; β = 0.782). Although higher factor loadings on the general factor were mainly related to repeated intrusive thoughts in the OCI, items in the PHQ and STAI were also observed, for example, “I feel inadequate” (item 35 in STAI. However, in the Japanese version, this item is translated as “melancholy” rather than “inadequate”; β = 0.751) and “Feeling bad about yourself – or that you are a failure or have let yourself or your family down” (item 6 in PHQ; β = 0.739). Factor loadings from the general factor to items in the SPQ, AQ, and ASRS were relatively small compared to those for the OCI, PHQ, and STAI, but showed moderate values (Fig 3a).

Specific factor labels were based on the content of items with factor loadings of  .30 or greater. Moreover, we referred to theoretical hypotheses, such as the higher-order spectra and symptom structures within the HiTOP framework, and subscales in questionnaires. For example, the factor that included “I tend to keep in the background on social occasions” (item 15 of SPQB) and “I am a good diplomat” (item 48 of AQ) was named “Detachment” based on the higher-order spectrum in the HiTOP framework. The other 10 specific factors were named “WellBeing,” “Mentalization,” “Checking,” “Ablutomania,” “ADHD,” “Overwhelmed,” “NumberPersistence,” “Bizarreness,” “Impatience,” and “Melancholy.” Factors such as “Melancholy” and “Detachment” showed relatively strong loadings across multiple questionnaires (i.e., transdiagnostic factors). Conversely, some individual questionnaires were divided into multiple factors, such as “Checking” and “Ablutomania” (i.e., factors corresponding to subscales of a particular questionnaire). Thus, joint factor analysis did not reproduce the original questionnaire units as specific factors but reorganized interpretable psychiatric symptoms into a hierarchical factor structure.

The factor correlation between “Checking” and “Ablutomania” was the strongest (0.384). Furthermore, “Detachment” had a relatively strong correlation with “Mentalization” (0.256). These correlations between factors not only demonstrate their relationships but also indicate the existence of intermediary factors nested within the hierarchy.

### Convergent and discriminant validity: Label-dependent analysis

To investigate the convergent and discriminant validity of the general and specific factors, the strength of associations with diagnoses was examined using the interpreted factor model described above. We again employed association strength, but this analysis provided a more detailed examination of the relationships between the interpreted specific factors and diagnostic categories. The results are presented in Fig 3b. The general factor showed significant discriminative power for all diagnoses (β = 0.810, p < 0.001 for any mental disorder; β = 0.527, p = 0.023 for schizophrenia; β = 0.636, p < 0.001 for mood disorders; β = 0.651, p < 0.001 for anxiety disorders, β = 1.048, p < 0.001 for obsessive-compulsive disorder; β = 1.164, p < 0.001 for neurodevelopmental disorder; and β = 0.563, p < 0.001 for unspecified disorders).

Conversely, each specific factor showed significant association strength only with particular diagnoses (Fig 3b). For example, “Melancholy” was significantly related only to the diagnosis of mood disorders (β = 0.223, p = 0.013), and “ADHD” was significantly correlated only with the diagnosis of neurodevelopmental disorders (β = 0.463, p = 0.028). Thus, the extracted specific factors were selective for conventional diagnostic categories and represented meaningful dimensions.

### Multi-layered hierarchy of psychiatric symptoms

Theoretical hypotheses, such as HiTOP, assume that psychiatric symptoms form a multi-layered hierarchy ranging from the general psychopathology factor and higher-order spectra to specific symptoms [5]. The results based on the oblique bifactor model with 12 factors suggest the existence of intermediary factors nested within this hierarchy. To further confirm this multi-layered hierarchy, we analyzed and visualized the overall hierarchical structure of psychiatric symptoms by integrating solutions across multiple levels, from few to several factors [12,32].

**Fig 4:**
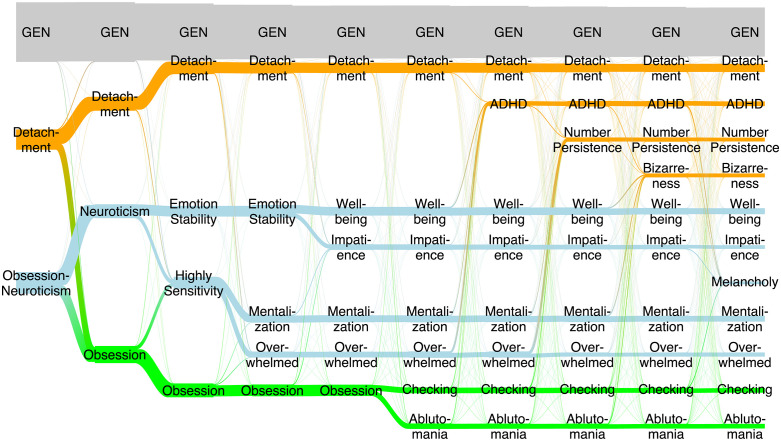
Multi-layered hierarchy of psychiatric symptoms. The heights of the panels with factor labels are plotted in proportion to the explained common variance (ECV). The thickness of the curves between these factors corresponds to the magnitude of the correlation coefficients between factor scores (a softmax function was used to highlight the size of the correlation coefficients for better visibility). Each column displays the results of factor analysis conducted with the same number of factors. The factors are reordered and assigned distinct colors, referring to the hierarchical structure of the Hierarchical Taxonomy of Psychopathology (HiTOP). Gen: general factor; ADHD: attention-deficit/hyperactivity disorder.

Fig 4 presents the visualization results. First, it is evident that the ECVs (indicated by the panel heights) of the general factors are substantial across the hierarchies, signifying that the general factors are stably and robustly extracted without the influence of specific factors.

Second, unlike the general factor, various specific factors were extracted depending on the number of factors. Furthermore, the order in which these factors appeared did not follow a consistent pattern from broad/abstract to narrow/specific components. For instance, in the 12-factor solution, we observed both broad factors (e.g., “Detachment”) and narrow factors (“NumberPersistence”). Even when the number of factors was small, several broad factors did not match the higher-order spectra in the HiTOP framework (e.g., “Obsession” and “HighSensitivity” in the five-factor solution), and lower-order specific factors corresponding to particular symptoms appeared (e.g., “Fatigue” in the six-factor solution). These observations present a counterexample to the clear hierarchy hypothesized in HiTOP.

Lastly, focusing on the progression of factors across hierarchies, specific factors may arise because of the branching of broader / more abstract factors. For example, “Mentalization” and “Fatigue” emerged from “HighSensitivity,” whereas “Checking” and “Ablutomania” originated from “Obsession.” In addition to branching, lower-order factors also arose due to the confluence of several factors, such as “ADHD” in the nine-factor solution and “Melancholy” in the 12-factor solution.

### Replication study

Similar analyses were applied to the two other datasets [17,33]. The results were largely similar to those of Dataset-1 (S1 Text). Regarding the dimensionality of Datasets-2 and -3, the number of factors could not be determined using conventional methods, and the ECV of the general factor showed high values, similar to Dataset-1 (Figs B and E in S1 Text). The trend where the ECV was primarily dominated by the general factor in Dataset-1 was also observed in Datasets-2 and -3 (Figs C, D, F, and G in S1 Text). However, unlike Dataset-1, the ECV exceeded  .7 when the number of factors was small in Dataset-2. Furthermore, the ECV in the higher-order factor model showed low values in Dataset-3, which was inconsistent with the results in Dataset-1. Similar to Dataset-1, omega reliability $\omega_{\text{t}}$ and omega hierarchical reliability $\omega_{\text{h}}$ of the general factor were sufficiently high, and $\omega_{\text{h}}$ of specific factors were low in Datasets-2 and -3 (Figs H–M in S1 Text). In Dataset-2, the correlation coefficients with diagnoses using the general factors were around  .25 regardless of the number of factors, and those with specific factors ranged around  .1–.2, similar to Dataset-1 (Figs N, O, and R in S1 Text).

Using the same procedures as for Dataset-1, solutions with 12 and 15 factors were selected for investigating interpretability in Datasets-2 and -3, respectively. Factor loadings on the general factors were higher for repeated and uncontrollable intrusive thoughts (OCI), followed by negative emotions (e.g., SDS, MCMI, and STAI), similar to the results for Dataset-1 (Figs P and Q in S1 Text). In the EFA, factors related to the subscales of the questionnaires were obtained, such as ablutomania and checking (OCI), withdrawal, spirituality, and bizarreness (SPQB), impulsivity and planning (BIS), and overwhelmed and impatience (STAI). Conversely, factors such as sleep-somatic (ISI and PHQ), hopelessness (PHQ and MCMI), well-being (SDS and STAI), and melancholy (SDS and STAI) were identified as transdiagnostic factors.

## Discussion

This study aimed to examine the psychometric properties of general and specific factors representing hierarchical psychiatric symptoms in the general population using item-level joint factor analysis. Due to the limited exploration of different types of hierarchical structures and varying numbers of factors in factor analysis, there is a lack of findings regarding the psychometric properties of general and specific factors in the general population. To address these limitations, we assessed and compared the reliability and validity of factor models across different types of hierarchical structures and numbers of factors.

The analysis revealed that the general factor had a stronger influence on the explained variance than the specific factors, although this influence was not strong enough to support unidimensionality. Similarly, internal consistency was adequate for the general factor but insufficient for the specific factors. Consistent with the *p*-factor concept, the general factor was associated with various diagnostic categories, although item loadings on the general factor primarily reflected internalizing symptoms and thought disorders. The concepts represented by specific factors became increasingly diverse and distinctive, owing to the branching and emergence of new factors as the number of factors increased. Moreover, several specific factors converged toward corresponding diagnostic categories, even after controlling for the effect of the general factor. In summary, this study revealed the distinct properties of both general and specific factors. The general factor demonstrated high reliability and convergent validity; however, its interpretability remains ambiguous. Conversely, the specific factors displayed high interpretability and reflected diverse psychiatric symptoms but showed low reliability.

Although several studies have sought to clarify the structure and properties of hierarchical psychiatric symptoms, our study had the advantage of performing a comprehensive item-level joint factor analysis in the general population. Most studies investigating the hierarchical structure of psychiatric symptoms rely on the presence or absence of diagnoses [9,10] or total scale scores [28]. These approaches have remained within conventional categorical frameworks because the units of analysis were restricted to traditional mental disorder diagnoses [8]. To overcome these limitations, several studies focusing on the general population have utilized item-level joint factor analysis; however, most did not assume a hierarchical structure [13, 14, 16-18]. Exceptional studies investigating reliability and validity have been limited in their exploration of different hierarchical structures and varying factor numbers [15,26,27,29,30].

Additionally, while previous research has predominantly focused on Western populations, our study targeted a non-Western (Japanese) sample. Consequently, our study confirmed that the properties of general and specific factors reported in previous research hold true across diverse cultural and ethnic backgrounds. Moreover, these results were replicated across three independently collected datasets.

The contributions of this study include: (1) identifying and reinforcing the psychometric properties and distinct roles of general and specific factors; (2) integrating and visualizing models with various factor numbers, providing empirical evidence for the hierarchical structure of psychopathology (e.g., HiTOP); (3) providing useful insights from an international perspective; and (4) highlighting the advantages and disadvantages of different tuning parameters through a comprehensive investigation of hierarchical structure and factor numbers, providing guidance for conducting hierarchical factor analysis.

### Properties of general and specific factors

We determined the psychometric properties of the general and specific factors. These findings were broadly consistent with previous research [19,26,29]. The low reliability of specific factors observed in this study serves as an example. Specifically, although the ECV and hierarchical omega reliability $\omega_{\text{h}}$ demonstrated good values for the general factor, those of the specific factors were poor regardless of the type of hierarchical structure and the number of factors. These results suggest that the variances were mainly explained by the general factor. Furthermore, the specific factors may not have sufficient internal consistency, and the total scores for each factor primarily depended on the general rather than the specific factors. These shortcomings have been reported in previous CFA studies [19,24]. Additionally, imbalances in dimensionality and internal consistency between the general and specific factors have been observed in many psychological and psychopathological tools [19,21]. This study reinforces these findings using item-level joint factor analysis with various tuning parameters and non-Western participants.

Despite the statistical weakness of the specific factors, this study elucidated their advantages. The relationships between the specific factors and diagnoses exhibited different patterns than those of the general factors. When these were analyzed independent of particular interpretations (the “Association Strength: Label-Independent Analysis” section), the factor scores of the general factors were strongly associated with diagnoses regardless of the number of factors. Conversely, the specific factor scores were unstable and weakly related to diagnoses (Fig 2d), suggesting that the specific factors were associated with particular diagnoses. To confirm this, we interpreted factor solutions with a particular structure and examined convergent and discriminant validity in detail (Fig 3b), finding that the specific factors constitute sufficiently concrete, meaningful factors. Moreover, the factor scores for the specific factors were associated with their corresponding diagnoses; for example, “Melancholy” was related to mood disorders. These results support the correspondence between the conventional categorical and alternative HiTOP approaches. In this way, while the general factor captures broad tendencies such as overall severity and common vulnerabilities, specific factors identify symptom clusters. This helps us better understand the diversity of individual phenotypes in psychopathology. In other words, focusing on specific factors allows us to understand symptom-specific mechanisms that might be overlooked by the general factor alone. Furthermore, the detailed symptom profiles provided by these specific factors complement conventional diagnostic systems and support the transition toward personalized treatment planning.

While the statistical properties of the general and specific factors were largely consistent across the three datasets, differences were observed in the granularity and labeling of certain specific factors. For instance, in Dataset-1, ADHD-related symptoms formed a single large “ADHD” factor that remained stable regardless of the number of factors. In contrast, Dataset-3 exhibited finer granularity, with ADHD-related symptoms splitting into “Impulsivity” and “Planning,” and depression-related symptoms splitting into “Decisiveness” and “Melancholy.” These discrepancies may stem from several factors. First, the evaluation scales differed (e.g., PHQ and ASRS in Dataset-1 vs. SDS and BIS in Dataset-3), which likely influenced the item-level coverage of specific domains. Second, differences in participant characteristics, such as the inclusion of clinical populations, may have affected symptom clustering. Furthermore, the lower reliability inherent in specific factors compared to the general factor may make them more sensitive to the structural nuances of each dataset. Whether these differences in granularity arise from the nature of the samples or the specific measurement tools remains a topic for future research.

Additionally, we examined the relationship between factor scores and computational phenotypes (i.e., individual attributes estimated from behavioral data in decision-making tasks using computational models; S1 Text), finding that specific factors were correlated with computational phenotypes in addition to the general factor (Figs U–Z in S1 Text). For example, “(In)Decisiveness” was significantly associated with the learning rate parameter in the reward-seeking task, whereas “Spirituality” was related to the inverse temperature parameter in the loss-avoidance task. As evidenced by these results, general and specific factors exhibit different patterns of relationships with external criteria depending on the context. Although the general factor is useful for providing an overview of an individual’s psychopathology and its severity, it does not fully describe the diversity of mental disorders. Conversely, multiple specific factors are useful for ideographically describing psychopathological profiles in detail.

While the interpretability of the specific factors was sufficient, caution is required when interpreting the general factor [22,23]. Specifically, whether the general factor, as statistically extracted in this study, genuinely reflects the theoretical construct known as the “general psychopathology factor” (*p*-factor) requires further investigation. Indeed, factor loadings of the general factor were relatively high across all questionnaires. However, items related to repeated intrusive thoughts in the OCI and depression in the PHQ and STAI were relatively higher than other items. This suggests the possibility that a common latent factor between internalizing symptoms and thought disorders was extracted, rather than the theoretical construct of the *p*-factor. Indeed, a previous study found that factor loadings related to internalizing symptoms were relatively high [7], and the content of the general factor has been interpreted in relation to various negative concepts [22]. The general factor estimated in this study could potentially be interpreted as reflecting another clinical psychological construct. Future studies should further investigate whether the estimated general factor represents constructs other than the *p*-factor.

### Hierarchy of psychiatric symptoms

A contribution of this study is the construction of hierarchical structures for psychiatric symptoms using item-level joint factor analysis. The factor analysis based on the 12-factor model did not merely reproduce the original questionnaire units as specific factors but instead reorganized the psychiatric symptoms. For example, “Melancholy” and “Detachment” were observed across several questionnaires (i.e., transdiagnostic symptoms), whereas certain questionnaires encompassed multiple latent factors, such as “Checking” and “Ablutomania” in the OCI (i.e., factors related to subscales). Furthermore, some specific factors were correlated with each other in the 12-factor model, suggesting the existence of intermediary factors nested within the hierarchy.

Consequently, we constructed and visualized a multi-layered hierarchy of psychiatric symptoms by integrating factor models from the bottom layer (larger number of factors) to the top layer (smaller number of factors). This visualization was partially consistent with the theoretical framework of HiTOP; for example, the specific factors at the higher-order level separated into several specific factors at the lower-order level. Additionally, the results revealed that the hierarchical structure proposed by the HiTOP framework does not necessarily align with empirical data in all aspects. For instance, in the 12-factor solution, we observed both broad factors (“Detachment”) and narrow factors (“NumberPersistence”).

This study identified diverse factors within the hierarchy of psychiatric symptoms, whereas previous studies utilized fewer factors and identified a limited hierarchy [15,27,30]. Similar to our analysis, Forbes et al. (2021) built a hierarchical structure using a bottom-up approach through sequential factor analysis and found that the empirically constructed hierarchy had both commonalities and differences with the theoretical structure proposed by HiTOP [12]. Although our procedure and results share several similarities with those of Forbes et al. (2021), we generated more detailed and granular factors. This can be attributed to our use of individual questionnaire items as the unit of analysis, whereas Forbes et al. (2021) used symptom-level scores by grouping several items. Additionally, we employed an oblique bifactor model, allowing us to identify specific factors at each level of the hierarchy while controlling for the influence of a general factor. As more research accumulates, we anticipate refining theoretically proposed hierarchical structures, such as HiTOP, on a more empirical basis.

### Cultural differences

Because few studies have analyzed the hierarchical structure of psychiatric symptoms in non-Western populations, this study provides useful insights from an international perspective. Psychiatric disorders are known to vary across cultures and ethnic groups; for example, Japan has been reported to have a lower prevalence of externalizing symptoms compared to the United States [47,48]. Such differences in prevalence may alter the co-occurrence of psychiatric symptoms, which in turn could influence the hierarchical factor structure. Since Dataset-2 included a questionnaire on externalizing symptoms (i.e., alcohol use), it was expected to reflect these differences, thereby allowing us to partially examine this hypothesis. Nevertheless, even though a scale for alcohol use was included, our findings showed that the general factor had higher reliability than the specific factors, similar to previous research. This suggests that cultural or ethnic differences in the prevalence of specific symptoms, such as alcohol use, may not strongly affect the reliability of the general factor.

Focusing on the content of the estimated factors, previous studies using the oblique model with three factors have reported that Japanese populations exhibit factor structures similar to those in Western populations [17,18]. However, it remains unclear whether this similarity holds when using models with more factors and including hierarchical structures. This uncertainty stems from the fact that, to our knowledge, very few studies have used such a wide range of questionnaires and applied item-level joint factor analysis as we did in this study. Future research is needed to explore in greater detail the types of factors that emerge and how they relate to cultural and ethnic backgrounds.

### Selection of hierarchical structures and factor numbers

Although EFA has various tuning parameters, there are no clear tuning strategies specific to the hierarchical structure of psychiatric symptoms in the general population. For example, Gillan et al. (2016) utilized oblique factor models [13], Polek et al. (2018) utilized bifactor models [27], and Gagne et al. (2020) utilized higher-order factor models [15]. Because this study exhaustively compared various types of hierarchical structures and factor numbers, our findings indicate the advantages and disadvantages of different tuning parameters. These insights can serve as guidance for future research using hierarchical factor analysis.

First, this study compared a wide range of factors to complement previous research that used fewer factors [13,27]. The results suggest that the number of factors should be determined based on the abstraction level and statistical properties of the specific factors of interest. We found that inconsistent results from standard EFA methods (e.g., scree plots, MAP criterion, and parallel analysis) make it challenging to clearly determine the number of factors using statistical methods alone. Our comprehensive analysis indicated that as the number of factors increases, latent factors implicit in a small number of factors may emerge. This aligns with previous findings that psychological and psychiatric constructs become more segmented as the number of factors increases [12]. This suggests that because certain factors only emerge with a larger number of factors, the optimal number must be determined based on research objectives and target constructs. However, it is important to acknowledge the trade-off between reliability (internal consistency) and the specificity of factors, because reliability tends to decrease as the number of factors increases. From a reliability standpoint, it is advisable not to increase the number of factors beyond what is necessary. If the focus is solely on the general factor, it appears that the number of factors has little influence because the ECV and internal consistency remain high [21,26,29].

Second, this study investigated different types of hierarchical structures (rotation methods), finding the oblique bifactor model convenient for analyzing the general factor rather than elucidating the hierarchical structure. Whether a bifactor or higher-order factor model is a better structure for psychiatric symptoms remains an ongoing debate [22,24]. Nevertheless, the differences in psychometric properties (such as ECV, $\omega_{\text{t}}$, and $\omega_{\text{h}}$) between the types of hierarchical structures were small in this study. Thus, it is challenging to clearly prioritize one type of hierarchical structure over another. However, bifactor models are convenient for exploring relationships with external criteria because the general and specific factors in higher-order factor models may exhibit potential collinearity. In particular, the oblique bifactor model is useful for detecting the presence of intermediate higher-order factors because it allows for correlations between specific factors. Additionally, because the ECVs in the hierarchical models were high, the factor loadings of the general factors were robust, even in the presence of model misconfigurations [21]. Therefore, if only the general factor is of interest rather than specific theoretical assumptions regarding model structures, the oblique bifactor model is sufficient from a practical standpoint.

### Limitations

This study has several limitations that should be addressed in future research. First, although the analyzed datasets were collected independently based on different objectives, there is a risk of sampling bias. For example, all datasets were obtained using crowdsourcing platforms in Japan, and the participants included more women than men. Additionally, the sample size may have been insufficient for models involving a large number of factors. In such complex models, the high number of parameters can lead to statistical instability, potentially compromising the interpretability of the results. Future studies should include more diverse and larger populations to investigate reproducibility. Second, temporal stability, such as test-retest reliability, was not investigated. Because comorbidities are likely to change over time, specific factors are expected to be less stable than the general factor. Third, the questionnaires and diagnoses were self-reported, and biases related to subjective judgments may have been included. This might explain the lack of correspondence between some specific factors and diagnoses. The relationships between the factor scores of general and specific factors and external criteria require further investigation. Building a predictive model that incorporates temporal fluctuations rather than relying solely on simple correlations from cross-sectional designs would be advantageous.

Finally, because the questionnaires included in the datasets did not cover all psychiatric symptoms (e.g., mania, somatization, and antisocial behavior), future studies should utilize a more diverse combination of instruments. Items in the OCI had relatively high factor loadings on the general factor in all datasets. This may be partly due to the properties of the OCI scale, which contains a relatively large number of items. Whether this observation is reproducible with other questionnaire combinations should be investigated. Such efforts may contribute to the construction of a more detailed and specific hierarchical structure of psychiatric symptoms. Additionally, using various combinations of questionnaires could help clarify the discrepancies observed in replication studies. These discrepancies include findings that the ECV with a small number of factors in Dataset-2 was slightly higher than that in the other datasets, that the ECV in the higher-order factor model in Dataset-3 decreased, and that the extracted specific factors differed between datasets. Regarding the first point, because Dataset-2 covers a wider range of symptoms than the other datasets, the result seems to contradict the idea that a single factor should have lower explanatory power when a dataset covers a broader range of symptoms. However, this contradiction can be resolved if individual items are, on average, explained more by the general factor than by specific factors. In fact, the additional items included symptoms related to dissociative disorders and borderline personality, which have a high affinity with the general factor identified in this study. Therefore, these items likely enhanced the overall explanatory power of the general factor. However, this study could not fully clarify the underlying mechanism, and this remains an important topic for future research.

## Conclusion

In this study, we investigated the psychometric properties of general and specific factors using item-level joint factor analysis. The analysis revealed the distinct roles of these factors. The general factor demonstrated high reliability and convergent validity, although its interpretability remained uncertain. Conversely, specific factors showed high interpretability and reflected diverse psychiatric symptoms, despite having low reliability. These distinct roles are expected to contribute to the development of alternative diagnostic systems for mental disorders and to idiographic descriptions of individuals.

## Supporting information

S1 Text Supplementary Material. This file contains all supplementary information, including the methods, results, and figures/tables referenced in the main text.
(DOCX)
