# Supplementary material for: Psychometric properties of hierarchical psychiatric symptoms on the general population
Source: PLOS Ment Health. 2026 Jun 17;3(6):e0000633. doi: 10.1371/journal.pmen.0000633 (PMC13274908; doi:10.1371/journal.pmen.0000633)
Supplement: S1 Text — This file contains all supplementary information, including the methods, results, and figures/tables referenced in the main text. (DOCX) [file pmen.0000633.s001.docx]

Supplementary Material:
Psychometric Properties of Hierarchical Psychiatric Symptoms on the General Population

# Demographic Data

Education status for Datasets-1 and -2 is provided in Tables A and B in S1 Text. Socioeconomic status was not obtained in these surveys. For demographic data (socioeconomic status and education level) of Dataset-3, please refer to the original publication [1].

# Reproduction Study

## Dimensionality

The results of the scree plot and parallel analysis for Dataset-1 are shown in Fig A in S1 Text.

In Dataset-2, the Kaiser-Guttman criterion, using the number of eigenvalues greater than one, suggested 25 factors. The BIC (Bayesian Information Criterion) did not achieve a minimum value by 50 factors. The MAP criterion suggested 12 factors, and parallel analysis suggested 19 factors (Fig B in S1 Text). The analyses were performed using factors ranging from 3 to 19. Figs C and D in S1 Text depict the explained common variances (ECVs) of the general and specific factors in Dataset-2, respectively.

In Dataset-3, the Kaiser-Guttman criterion suggested 25 factors, and the BIC did not achieve a minimum value by 50 factors. The MAP criterion suggested 19 factors, and parallel analysis suggested 22 factors (Fig E in S1 Text). The analysis was performed using factors ranging from 3 to 19. Figs F and G in S1 Text depict ECVs of the general and specific factors in Dataset-3, respectively.

As a supplementary analysis, Table C in S1 Text presents model fit indices for confirmatory factor analysis (CFA) models with varying numbers of factors. In the CFA, a factor model with all paths allowed was assumed. The TLI (Tucker–Lewis Index), RMSEA (Root Mean Square Error of Approximation), and BIC were computed based on models estimated using the maximum likelihood estimator with the “MLMV” option in the “lavaan” package. As shown in the table, models with a very large number of factors tended to be favored by the fit indices; however, such models sometimes included factors defined by only a single item, making them difficult to interpret. Although the BIC suggested models with relatively few factors in the CFA, inconsistencies in the number of factors proposed by different methods remained. Therefore, we did not rely heavily on fit indices when selecting the number of factors.

## Reliability

Figs H, I, and J in S1 Text depict the omega reliability $\omega_{t}$ and omega hierarchical reliability $\omega_{h}$ of the general factor, and omega hierarchical reliability $\omega_{h}$ of the specific factors in Dataset-2, respectively.

Figs K, L, and M in S1 Text depict omega reliability $\omega_{t}$ and omega hierarchical reliability $\omega_{h}$ of the general factor, and omega hierarchical reliability $\omega_{h}$ of the specific factors in Dataset-3, respectively.

## Association Strength

Figs N and O in S1 Text depict the association strength of the general and specific factors for diagnostic history in Dataset-2.

## Content Validity

The procedures used to investigate factor interpretation were applied to Datasets-2 and -3.

For Dataset-2, the 12-factor solution was supported (Fig P in S1 Text). The general factor loaded strongly on a wide range of items. However, the AUDIT items did not load strongly on the general factor. In Dataset-2, as a criterion for interpretation, less than four items exceeding .25 was adopted. The specific factors were labeled “Alcohol,” “Dissociation,” “Anger,” “Sleep-Somatic,” “Ablutomania,” “Withdrawal,” “Checking,” “Hopelessness,” “Spirituality,” “NumberPersistence,” and “Bizarreness.”

For Dataset-3, the 15-factor solution was supported (Fig Q in S1 Text). The general factor loaded strongly on a wide range of items. The specific factors were labeled “WellBeing,” “Impulsivity,” “Ablutomania,” “Restful,” “Planning,” “Withdrawal,” “Bizarreness,” “NumberPersistence,” “Impatience,” “Spirituality,” “Checking,” “Overwhelmed,” “Decisiveness,” and “Melancholy.”

## Convergent and Discriminant Validity

Fig R in S1 Text depicts the association strength of each factor for diagnoses.

## Multi-Layered Hierarchy of Psychiatric Symptoms

Figs S and T in S1 Text show the results of the hierarchical factor analysis using Goldberg’s method for Datasets-2 and -3, respectively.

# Association with Computational Phenotypes

In Dataset-3 [1], participants performed two types of reinforcement tasks: reward-seeking and loss-avoidance tasks. We investigated the associations between factor scores and computational phenotypes estimated from their behaviors in these tasks using Pearson’s correlation coefficients. In this study, computational phenotypes such as learning rate, forgetting rate, inverse temperature, choice-trace decay rate, and choice-trace weight were estimated by fitting the behavioral sequences of each participant to the computational model.

Figs U, V, and W in S1 Text depict the association strength of the general and specific factors for computational phenotypes in the reward-seeking task in Dataset-3. Additionally, Figs X, Y, and Z in S1 Text depict the association strength of the general and specific factors for computational phenotypes in the loss-avoidance task in Dataset-3.

# References

1. Suzuki S, Yamashita Y, Katahira K. Psychiatric symptoms influence reward-seeking and loss-avoidance decision-making through common and distinct computational processes. Psychiatry Clin Neurosci. 2021;75(9):277-285. doi:10.1111/pcn.13279

# Supplementary Tables

Table A

Educational background in Dataset-1.

|  | Frequency |
| --- | --- |
| Junior high school | 20 |
| High school | 440 |
| University education | 642 |
| Graduate school | 61 |

Table B

Educational background in Dataset-2.

|  | Frequency |
| --- | --- |
| Junior high school | 18 |
| High school | 293 |
| University education | 615 |
| Graduate school | 59 |

Table C

Fit indices in the factor analysis.

|  | Dataset-1 | | | Dataset-2 | | | Dataset-3 | | |
| --- | --- | --- | --- | --- | --- | --- | --- | --- | --- |
| #Factor | TLI | RMSEA | BIC | TLI | RMSEA | BIC | TLI | RMSEA | BIC |
| 3 | 0.583 | 0.048 | 448590 | 0.574 | 0.057 | 400363 | 0.651 | 0.050 | 589302 |
| 4 | 0.636 | 0.045 | 443909 | 0.631 | 0.053 | 396514 | 0.695 | 0.047 | 582788 |
| 5 | 0.676 | 0.043 | 440607 | 0.677 | 0.050 | 393642 | 0.734 | 0.044 | 577194 |
| 6 | 0.700 | 0.041 | 438912 | 0.717 | 0.046 | 391300 | 0.759 | 0.042 | 573915 |
| 7 | 0.724 | 0.039 | 437366 | 0.741 | 0.044 | 390161 | 0.777 | 0.040 | 571801 |
| 8 | 0.742 | 0.038 | 436419 | 0.759 | 0.043 | 389514 | 0.794 | 0.039 | 569993 |
| 9 | 0.760 | 0.037 | 435561 | 0.773 | 0.042 | 389194 | 0.806 | 0.038 | 568885 |
| 10 | 0.776 | 0.035 | 434973 | 0.787 | 0.040 | 388917 | 0.818 | 0.036 | 567819 |
| 11 | 0.788 | 0.034 | 434683 | 0.800 | 0.039 | 388741 | 0.830 | 0.035 | 566800 |
| 12 | 0.798 | 0.034 | 434680 | 0.808 | 0.038 | 388909 | 0.839 | 0.034 | 566235 |
| 13 | 0.807 | 0.033 | 434752 | 0.816 | 0.037 | 389092 | 0.846 | 0.033 | 566046 |
| 14 | 0.816 | 0.032 | 434899 | 0.823 | 0.037 | 389328 | 0.853 | 0.033 | 565890 |
| 15 | 0.824 | 0.031 | 435179 | 0.830 | 0.036 | 389672 | 0.860 | 0.032 | 565777 |
| 16 | 0.831 | 0.031 | 435499 | 0.836 | 0.035 | 390052 | 0.866 | 0.031 | 565793 |
| 17 | 0.838 | 0.030 | 435883 | 0.841 | 0.035 | 390468 | 0.870 | 0.031 | 566063 |
| 18 | 0.844 | 0.029 | 436370 | 0.847 | 0.034 | 390929 | 0.876 | 0.030 | 566170 |
| 19 | 0.849 | 0.029 | 436994 | 0.850 | 0.034 | 391494 | 0.881 | 0.029 | 566407 |

*Note.* BIC: Bayesian Information Criterion; RMSEA: Root Mean Square Error of Approximation; TLI: Tucker–Lewis Index.

Table D

Descriptive statistics (mean and standard deviation) for the skewness and kurtosis of factor scores derived from oblique bifactor models.

|  | Dataset-1 |  | Dataset-2 |  | Dataset-3 |  |
| --- | --- | --- | --- | --- | --- | --- |
| #Factor | Skewness | Kurtosis | Skewness | Kurtosis | Skewness | Kurtosis |
| 3 | 0.47(0.82) | 1.22(1.76) | 1.38(0.24) | 4.32(1.84) | 0.72(0.71) | 1.53(1.33) |
| 4 | 0.43(0.75) | 1.22(1.53) | 1.61(0.53) | 5.34(2.45) | 0.45(0.78) | 1.34(1.14) |
| 5 | 0.39(0.71) | 1.13(1.53) | 1.54(0.58) | 5.36(2.88) | 0.37(0.72) | 1.14(1.13) |
| 6 | 0.21(0.66) | 0.94(1.26) | 1.37(0.69) | 4.71(3.30) | 0.57(0.54) | 1.03(1.13) |
| 7 | 0.27(0.71) | 1.16(1.34) | 1.19(0.86) | 4.22(3.37) | 0.49(0.56) | 1.05(0.98) |
| 8 | 0.34(0.69) | 1.32(1.26) | 1.09(0.91) | 3.95(3.35) | 0.53(0.51) | 1.19(0.94) |
| 9 | 0.23(0.69) | 1.26(1.21) | 0.96(0.97) | 3.57(3.61) | 0.44(0.51) | 1.11(1.02) |
| 10 | 0.25(0.64) | 1.14(1.23) | 1.05(0.83) | 3.75(3.51) | 0.45(0.51) | 1.08(1.06) |
| 11 | 0.25(0.60) | 1.01(1.23) | 0.99(0.79) | 3.52(3.39) | 0.42(0.48) | 1.08(1.00) |
| 12 | 0.24(0.58) | 0.99(1.05) | 0.95(0.76) | 3.35(3.19) | 0.50(0.54) | 1.14(1.04) |
| 13 | 0.26(0.56) | 0.98(1.04) | 0.90(0.74) | 3.41(2.98) | 0.48(0.54) | 1.16(1.00) |
| 14 | 0.24(0.56) | 0.89(1.04) | 0.60(1.10) | 4.12(4.06) | 0.52(0.51) | 1.23(1.03) |
| 15 | 0.27(0.55) | 0.87(0.96) | 0.50(1.10) | 3.91(3.87) | 0.45(0.53) | 1.24(1.13) |
| 16 | 0.28(0.53) | 0.87(0.95) | 0.73(0.82) | 3.23(2.60) | 0.42(0.55) | 1.20(1.05) |
| 17 | 0.26(0.53) | 0.87(0.87) | 0.58(1.03) | 3.87(3.81) | 0.41(0.55) | 1.15(1.11) |
| 18 | -- | -- | 0.55(0.99) | 3.67(3.67) | 0.39(0.54) | 1.12(1.17) |
| 19 | -- | -- | 0.55(0.97) | 3.58(3.64) | 0.40(0.53) | 1.15(1.14) |

# Supplementary Figures

Fig A

Results of parallel analysis in Dataset-1.


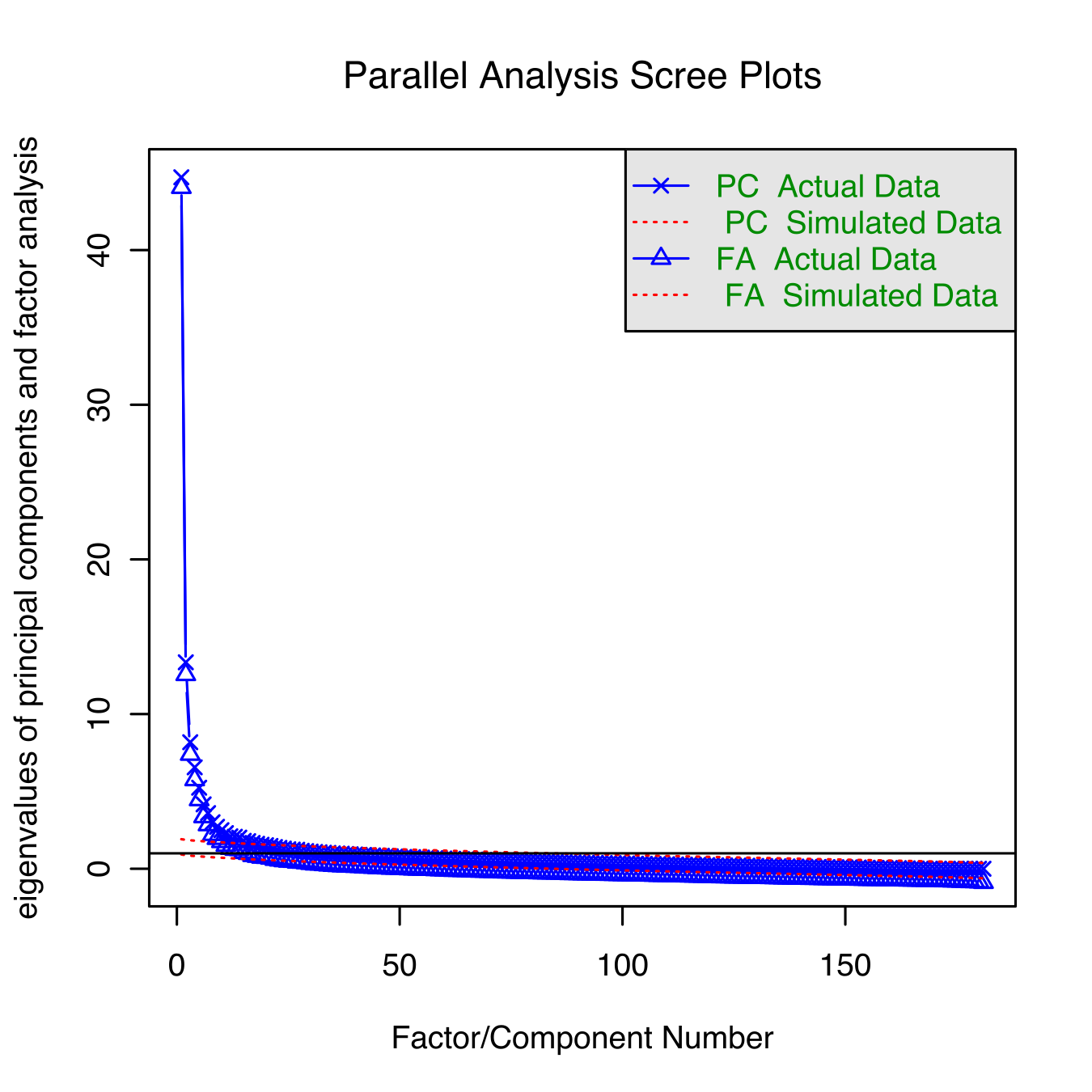


Fig B

Results of parallel analysis in Dataset-2.


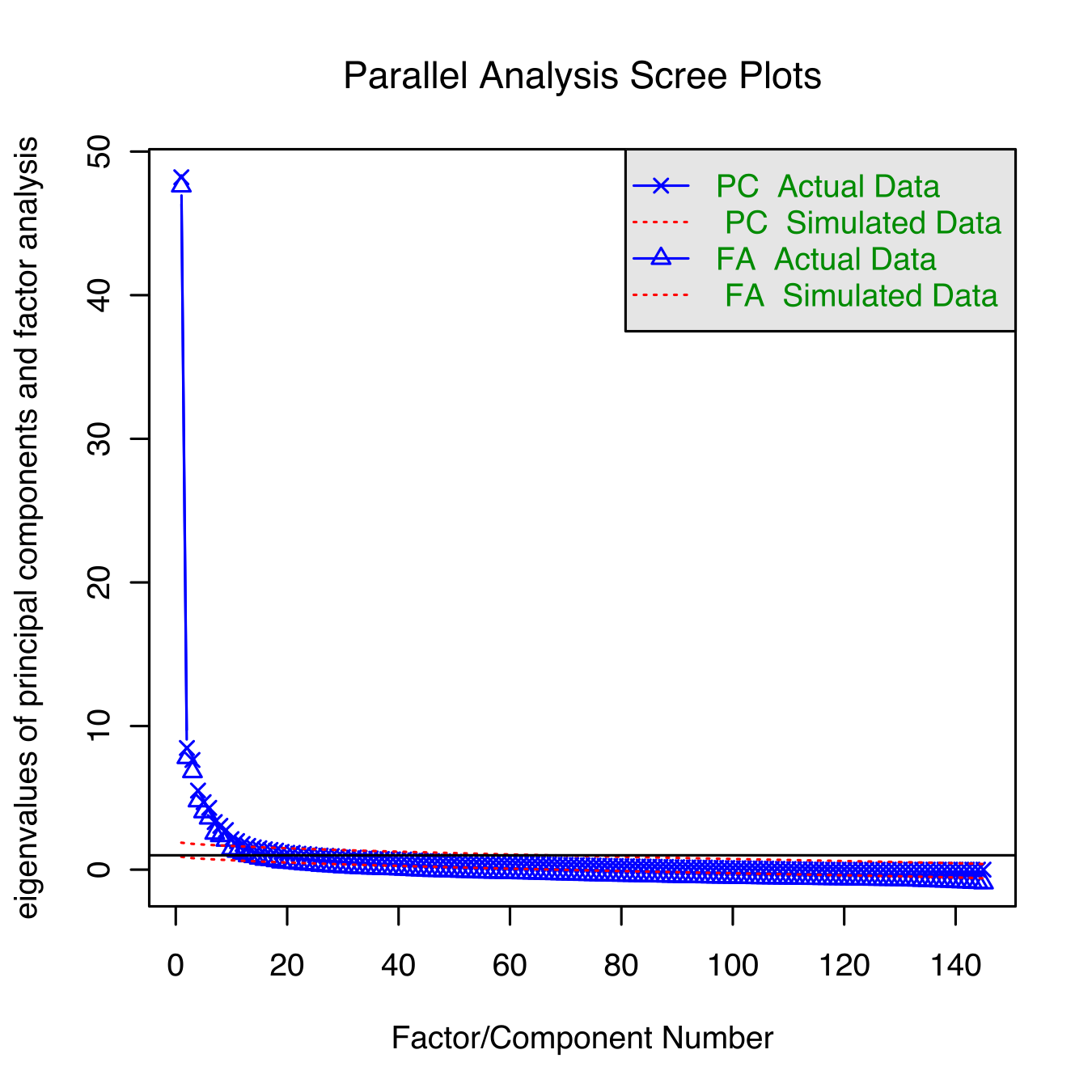


Fig C

Explained common variance (ECV) of the general factor in each factor model in Dataset-2.


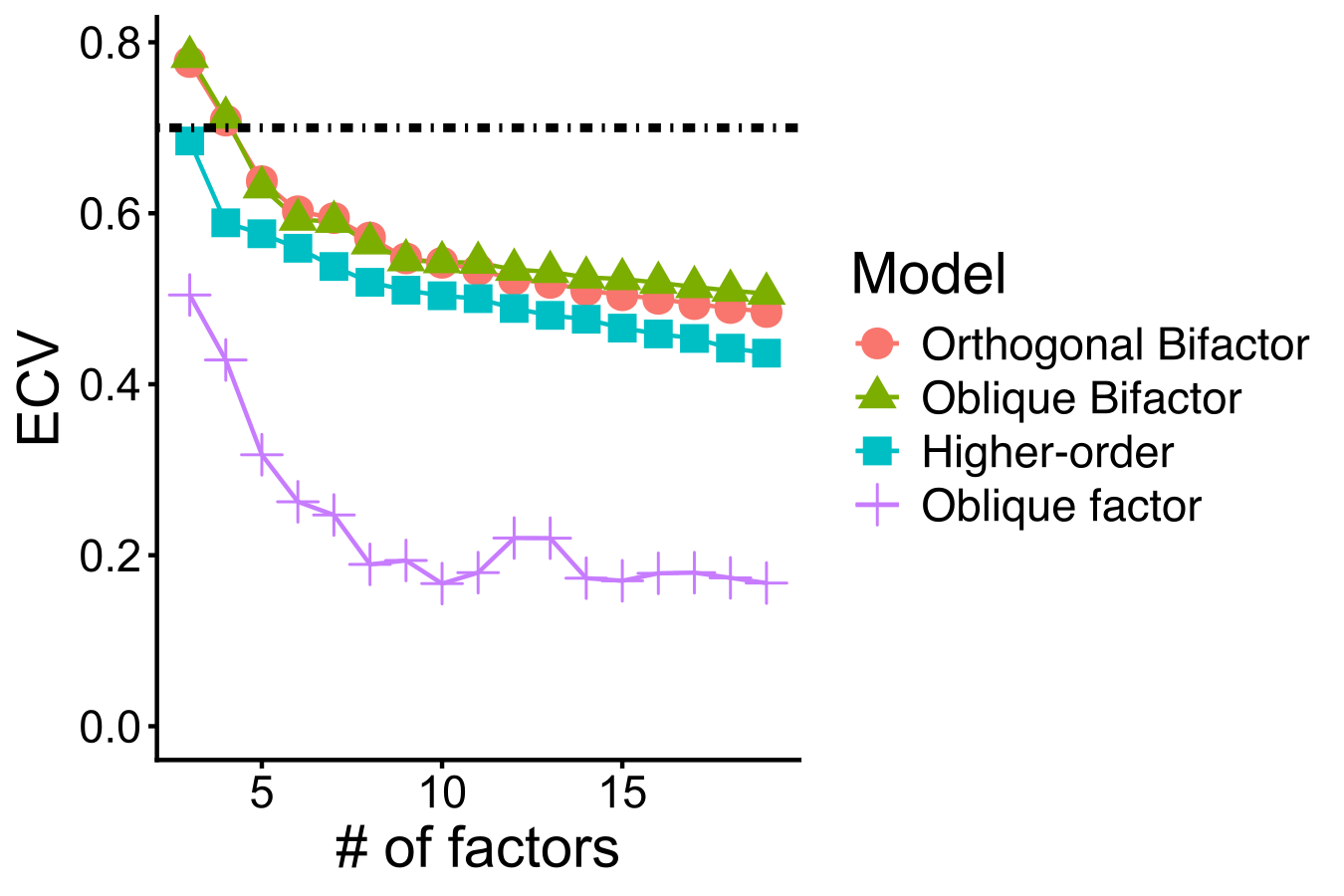


Fig D

Explained common variance (ECV) of the specific factors in each factor model in Dataset-2.


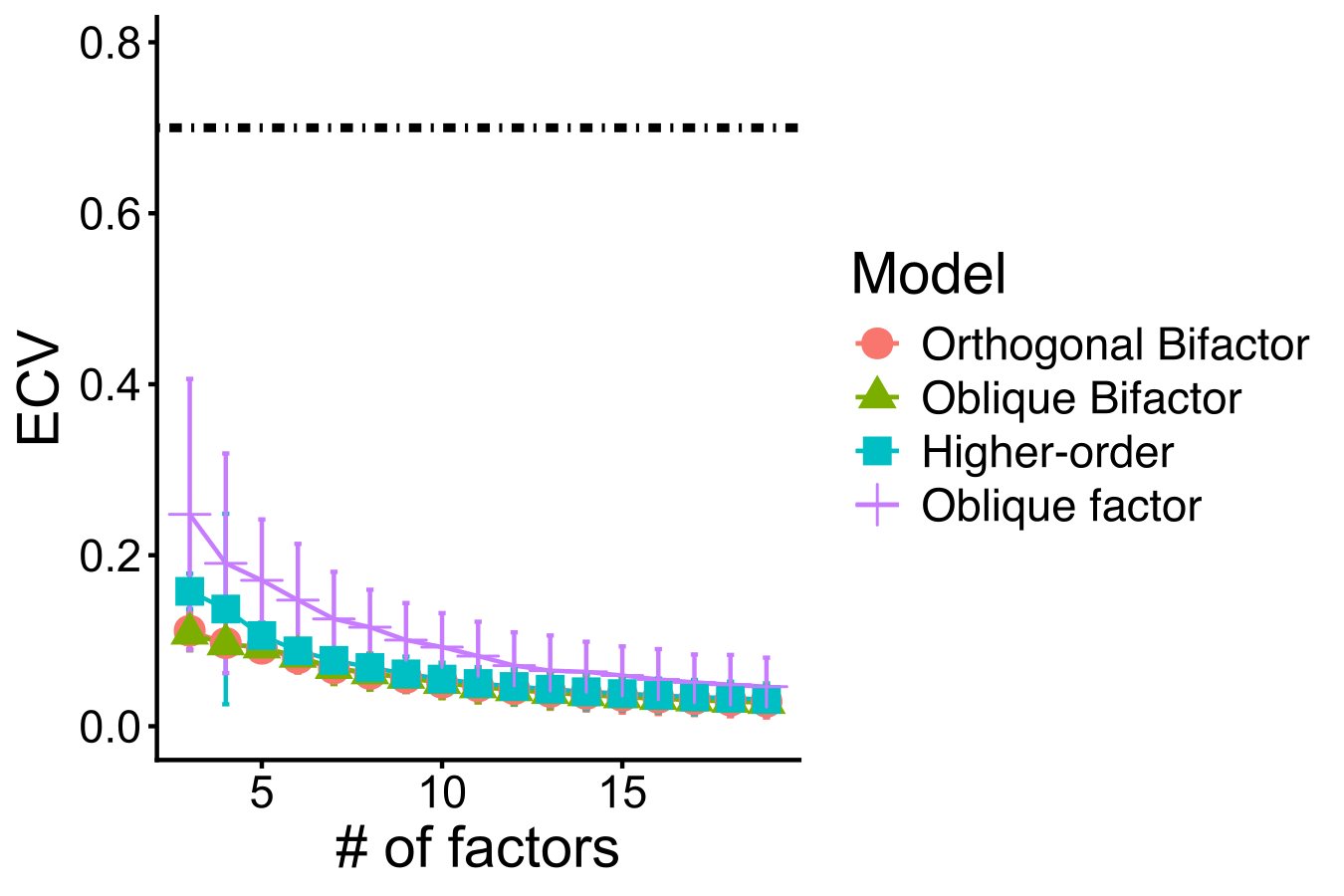


Fig E

Results of parallel analysis in Dataset-3.


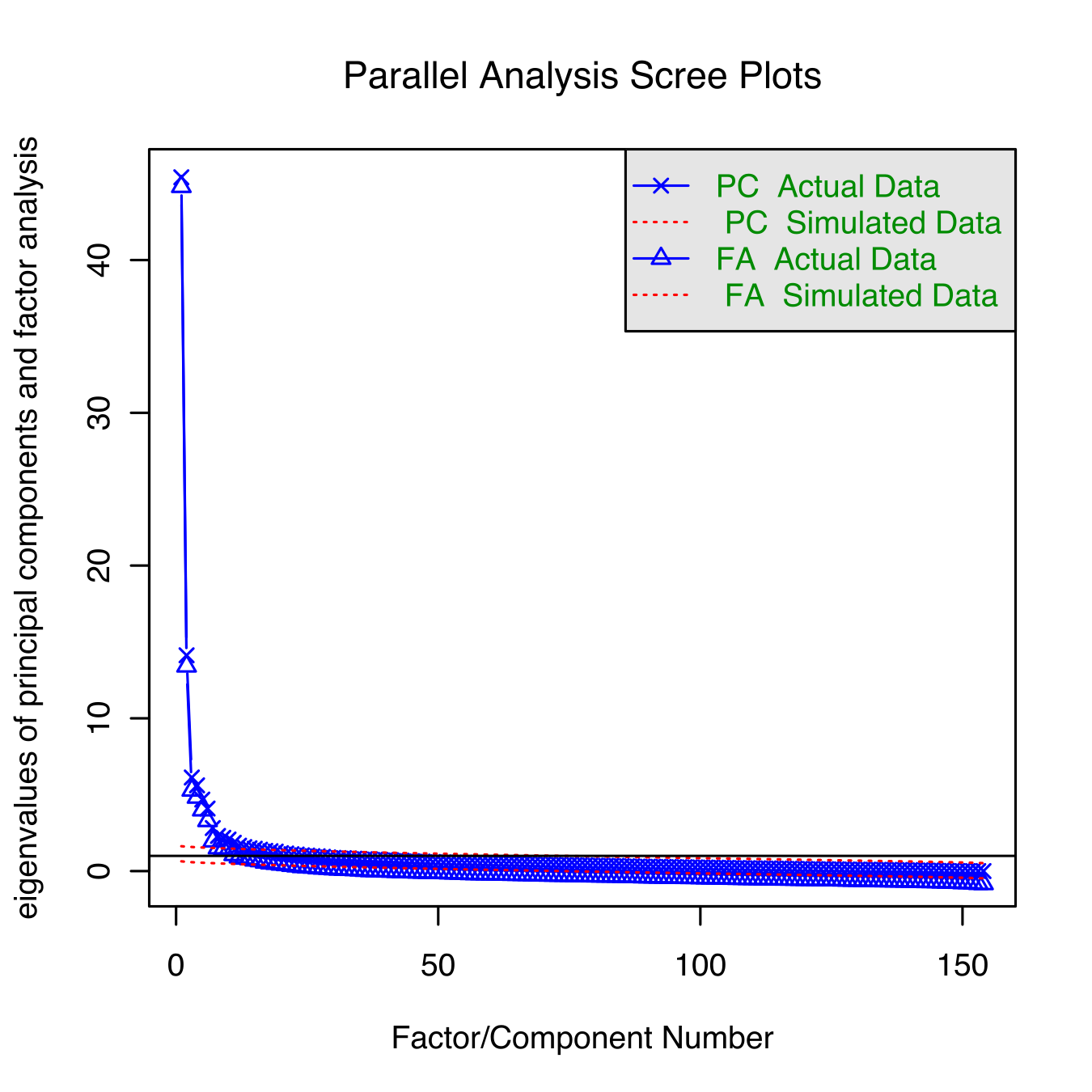


Fig F

Explained common variance (ECV) of the general factor in each factor model in Dataset-3.


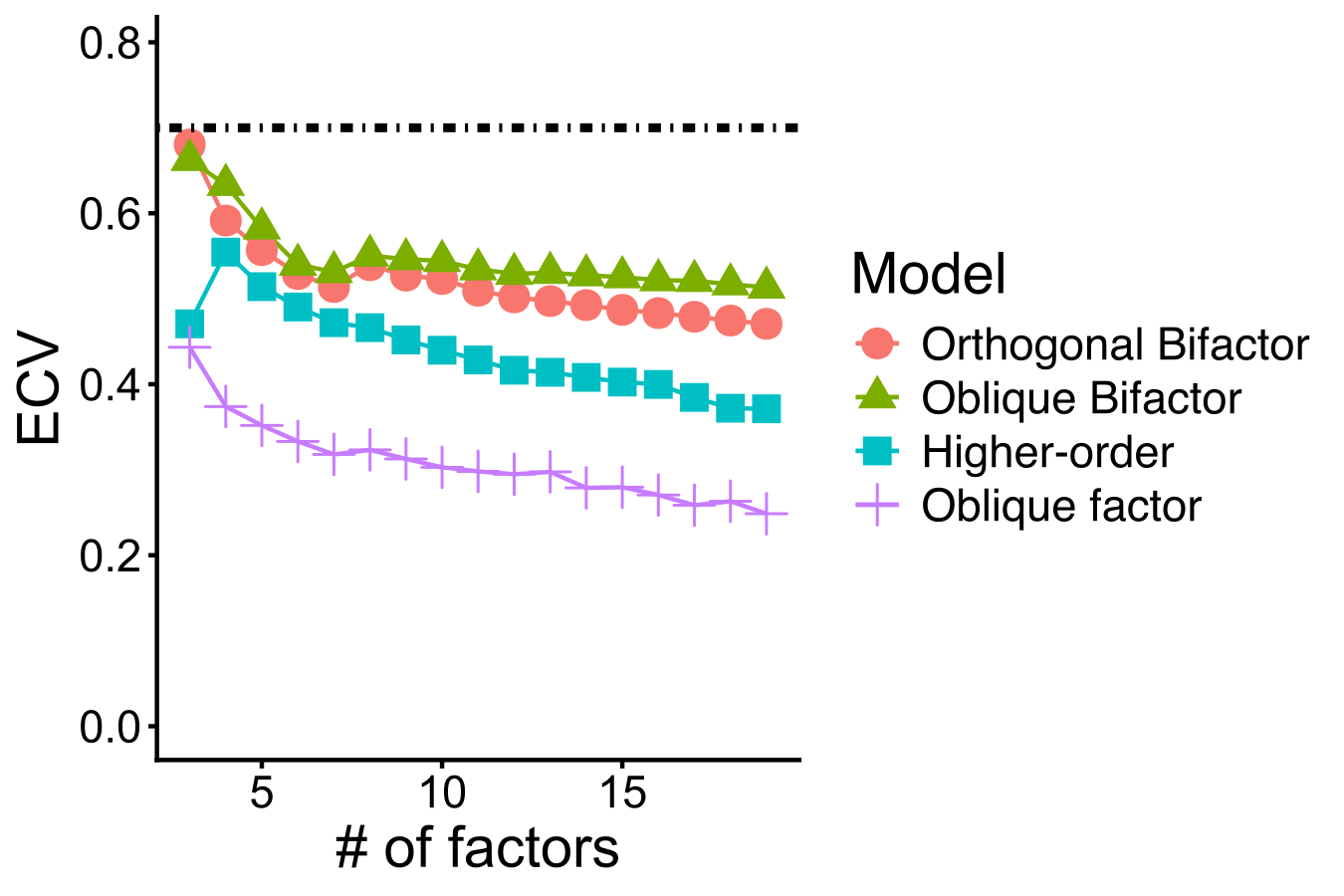


Fig G

Explained common variance (ECV) of the specific factors in each factor model in Dataset-3.


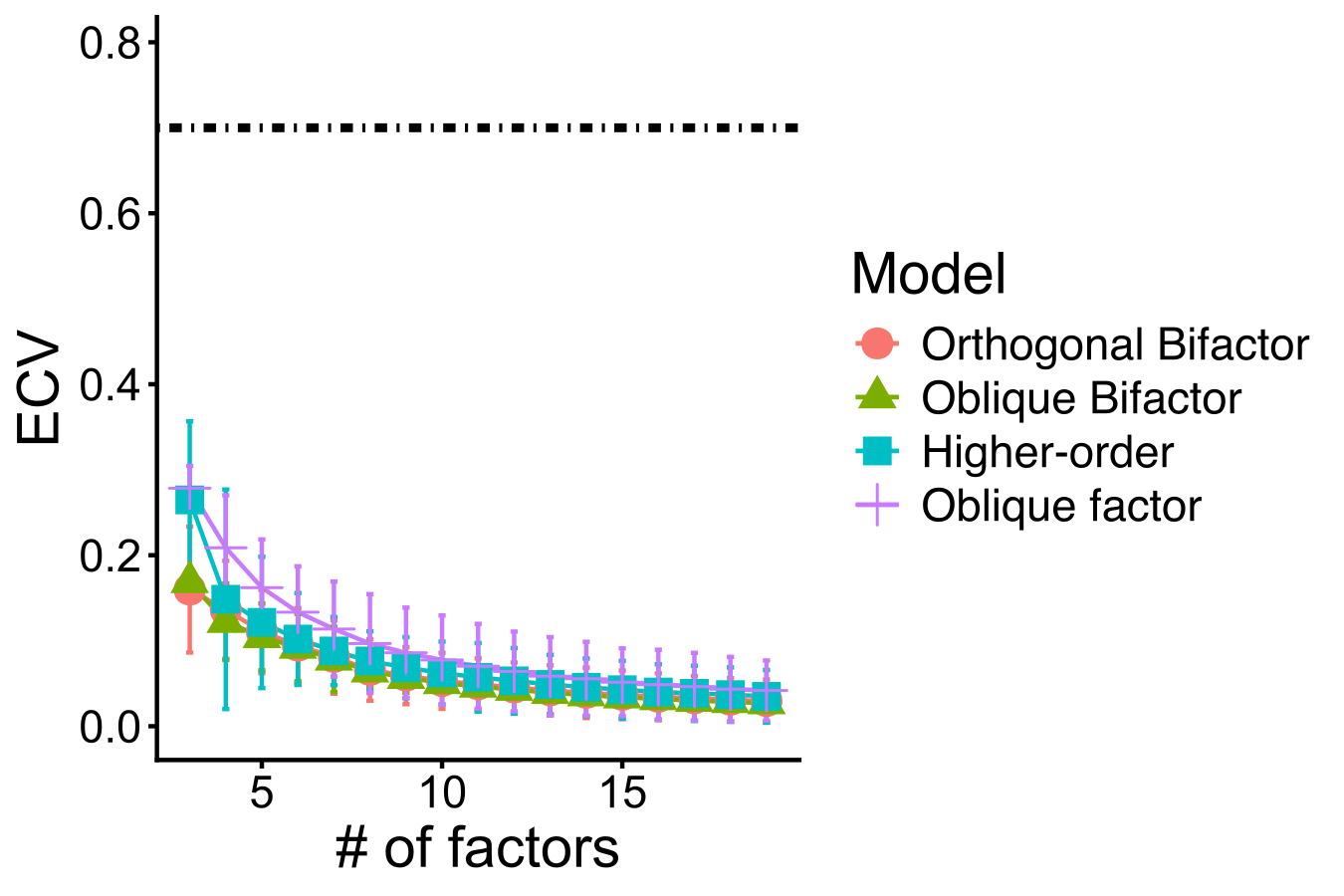


Fig H

Omega reliabilities $\omega_{t}$ in each factor model in Dataset-2.


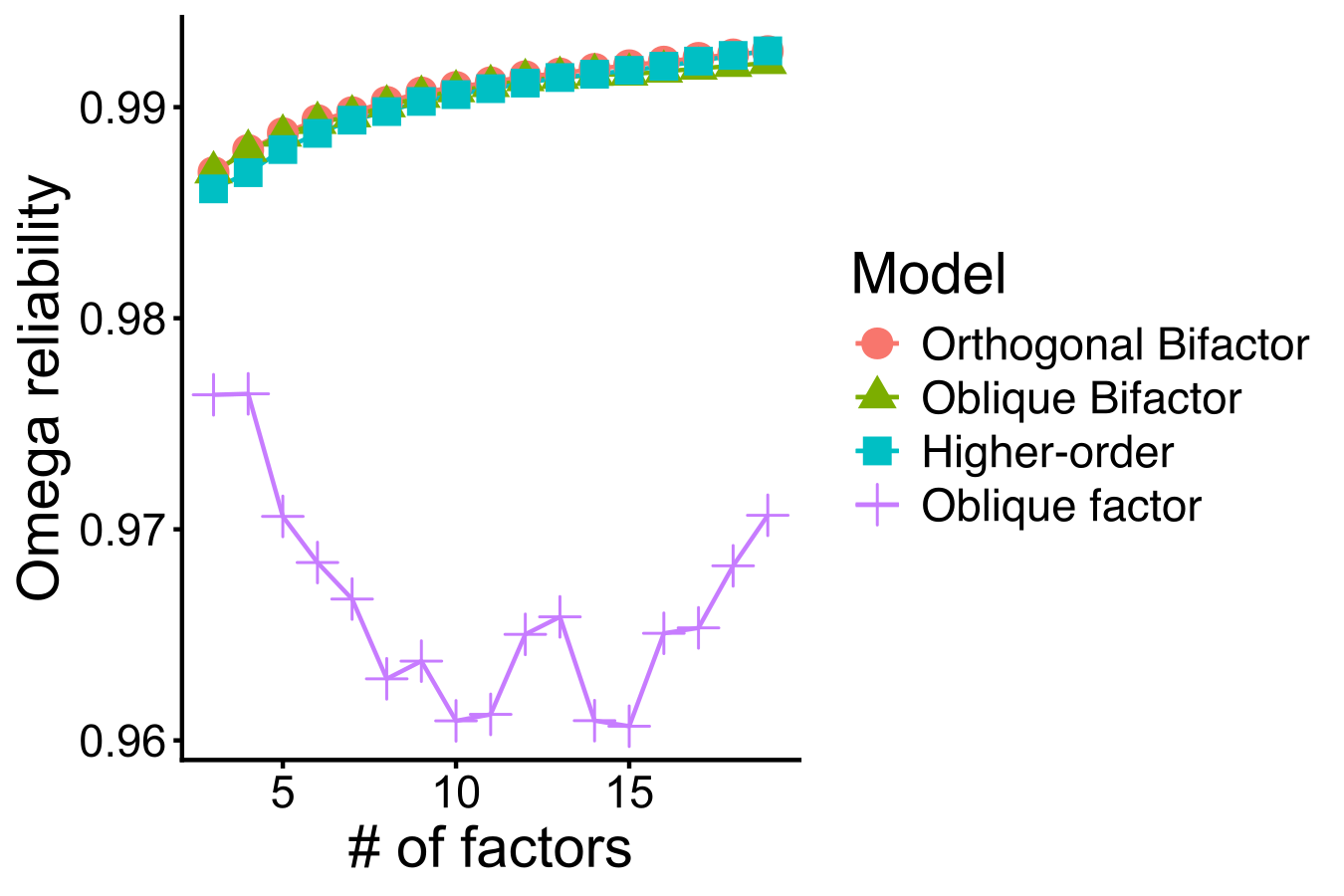


Fig I

Omega hierarchical reliabilities $\omega_{h}$ for general factors in each factor model in Dataset-2.


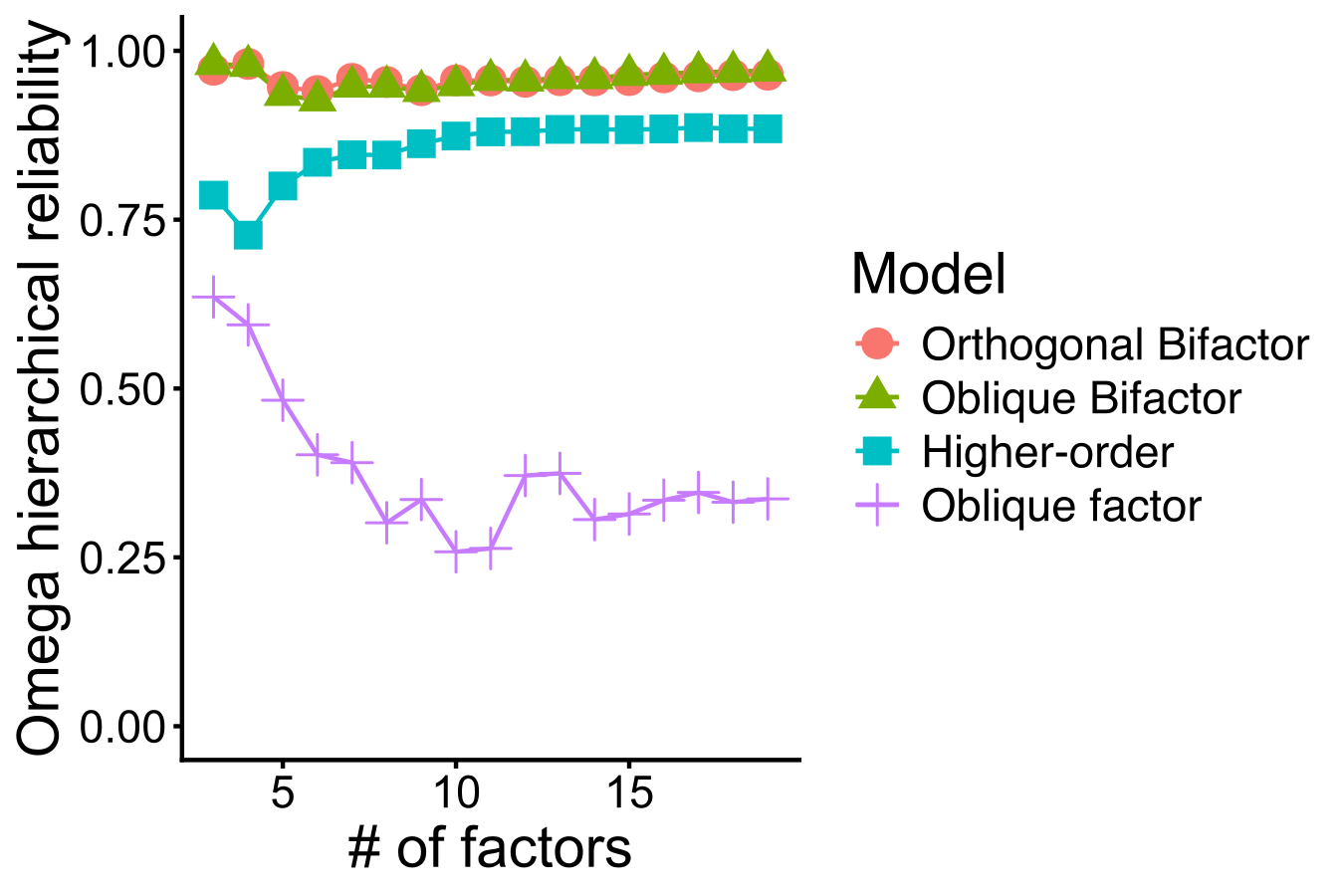


Fig J

Omega hierarchical reliabilities $\omega_{h}$ for specific factors in each factor model in Dataset-2.


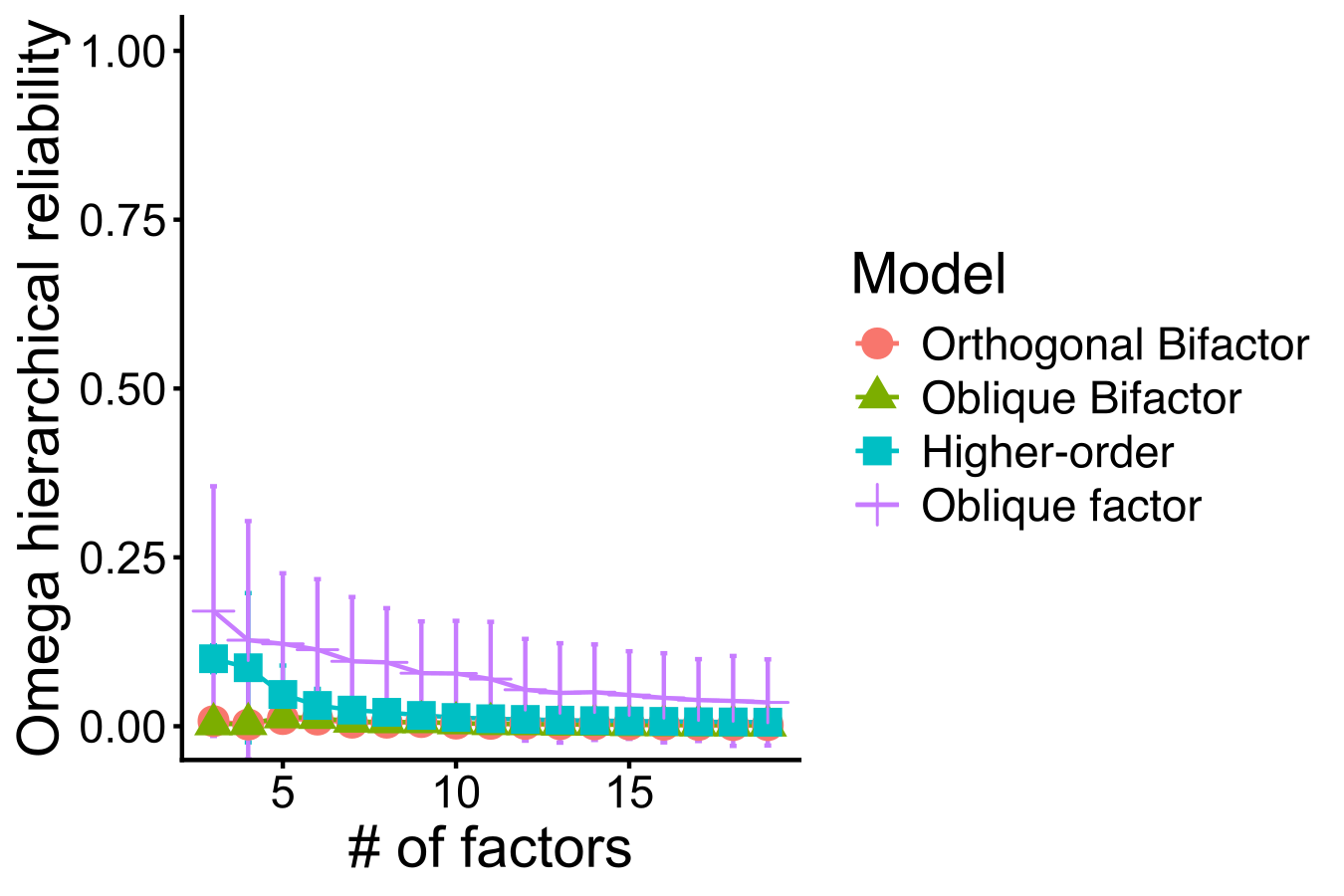


Fig K

Omega reliabilities $\omega_{t}$ in each factor model in Dataset-3.


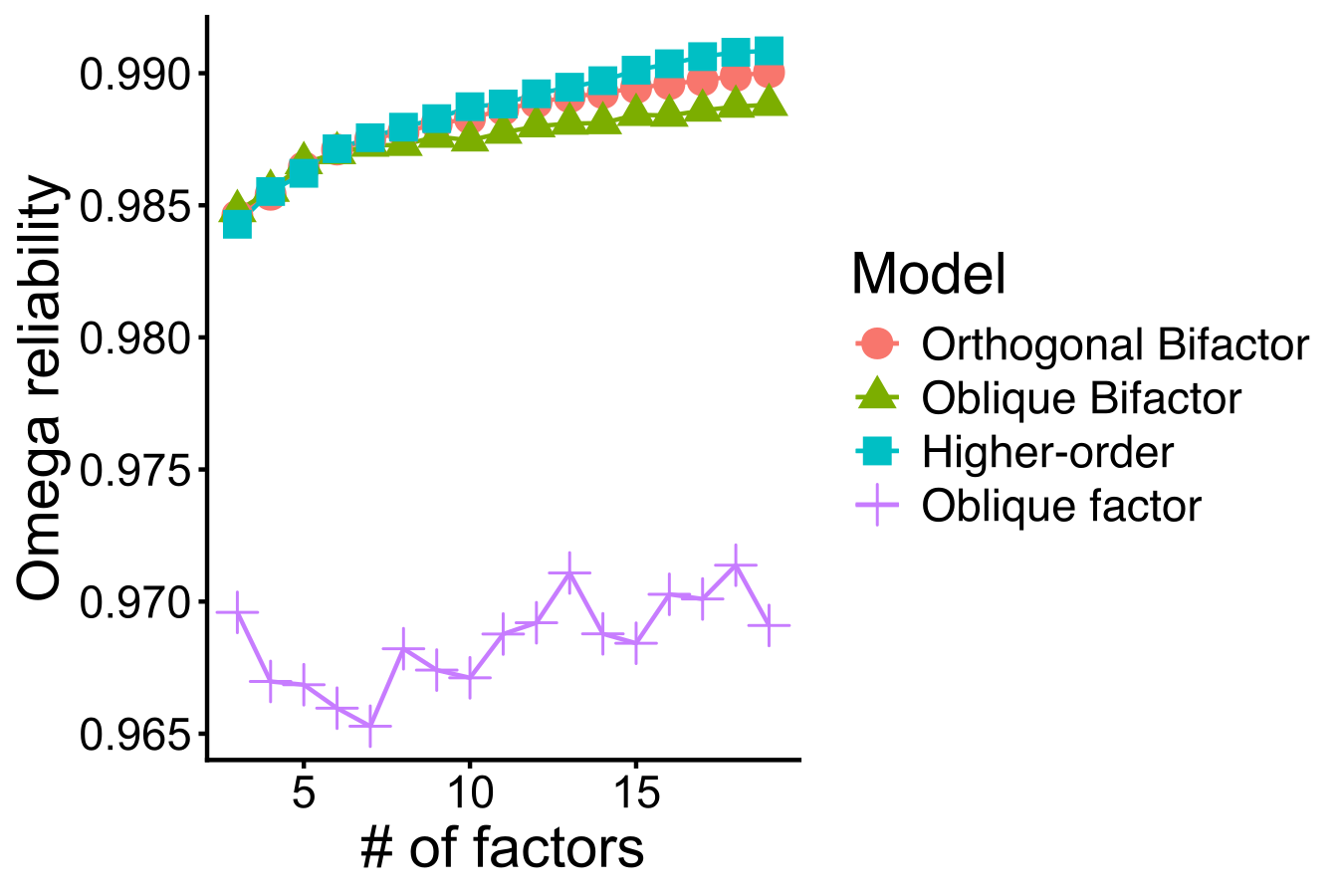


Fig L

Omega hierarchical reliabilities $\omega_{h}$ for general factors in each factor model in Dataset-3.


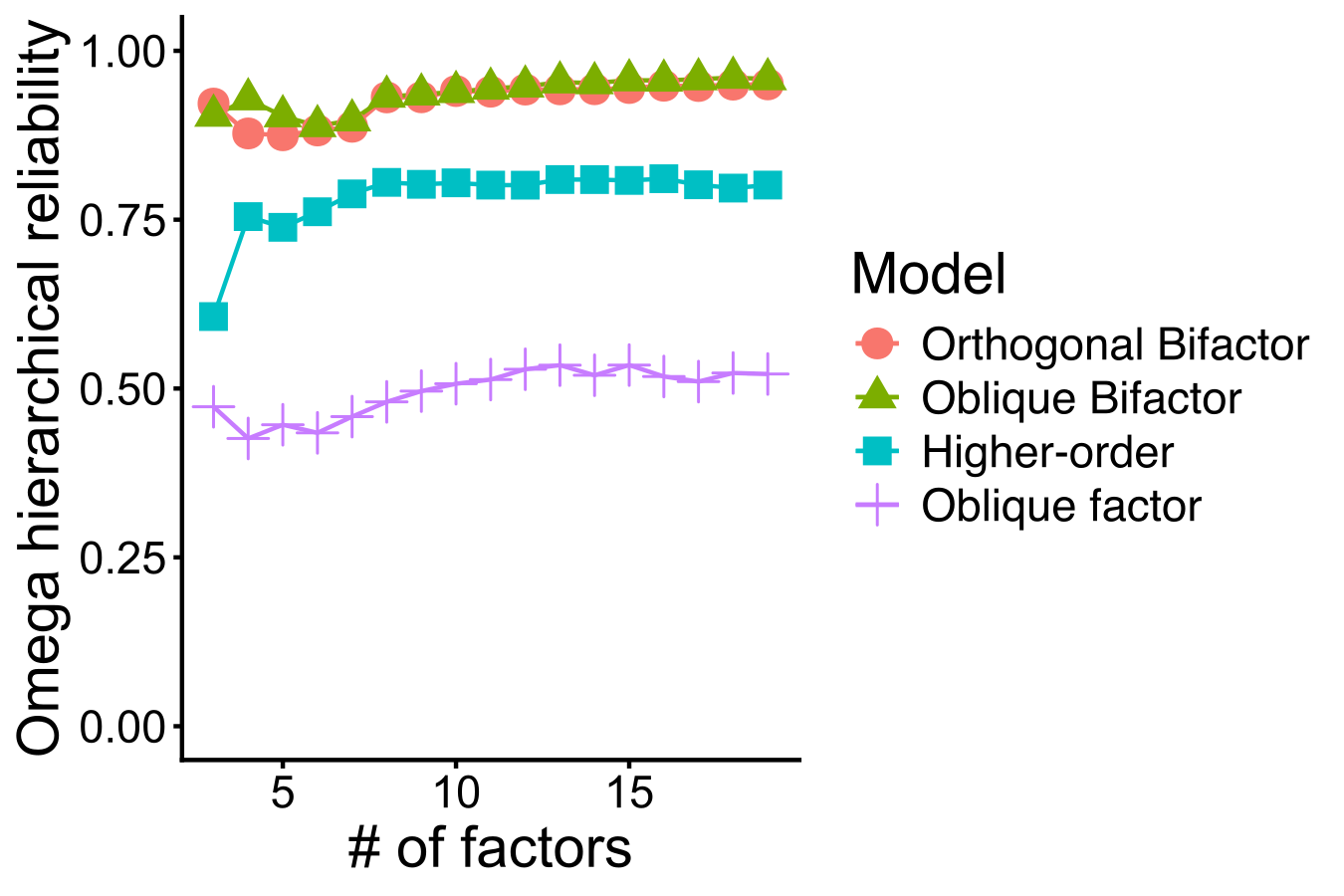


Fig M

Omega hierarchical reliabilities $\omega_{h}$ for specific factors in each factor model in Dataset-3.


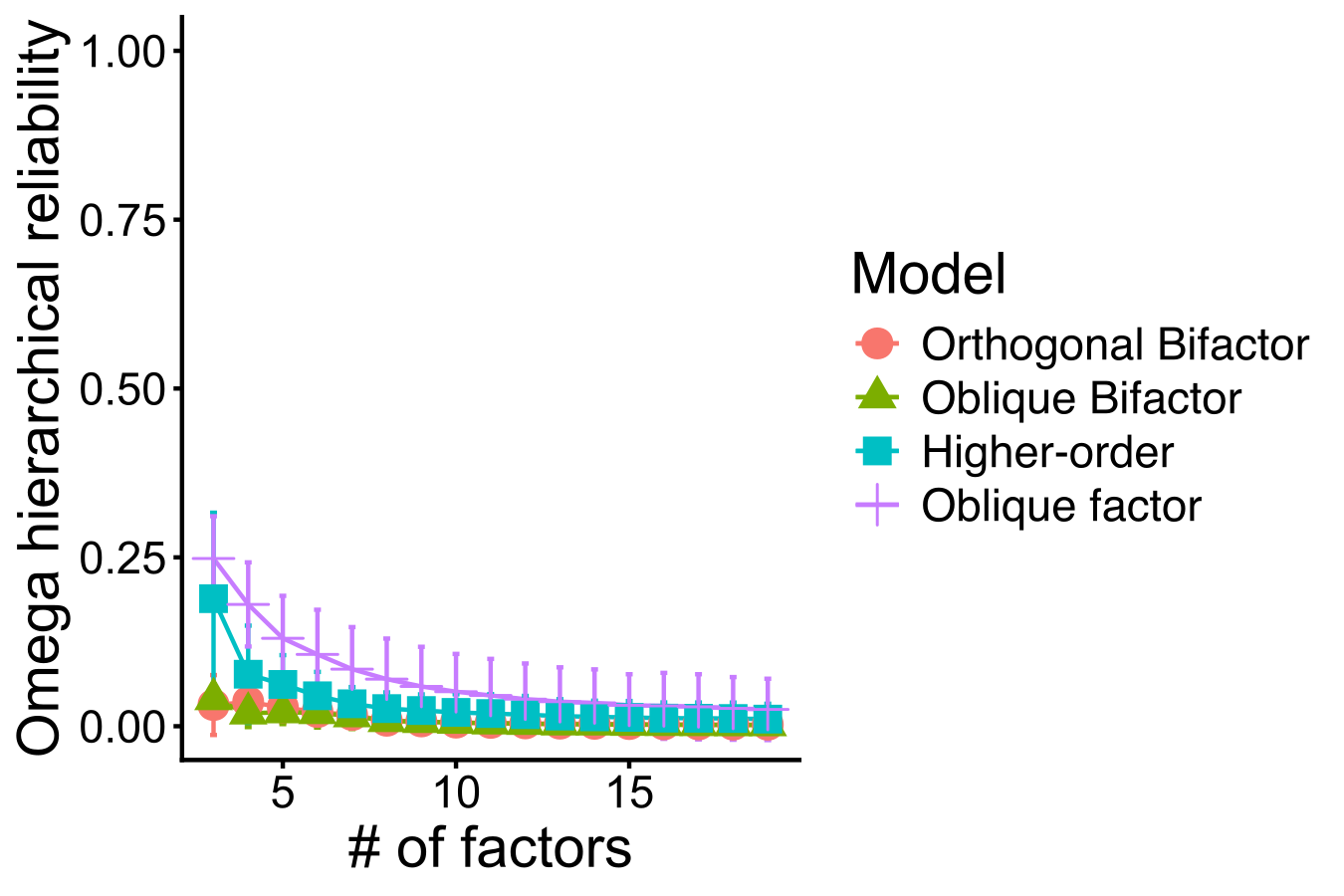


Fig N

Association strength of the general factors for diagnostic history in each factor model in Dataset-2.


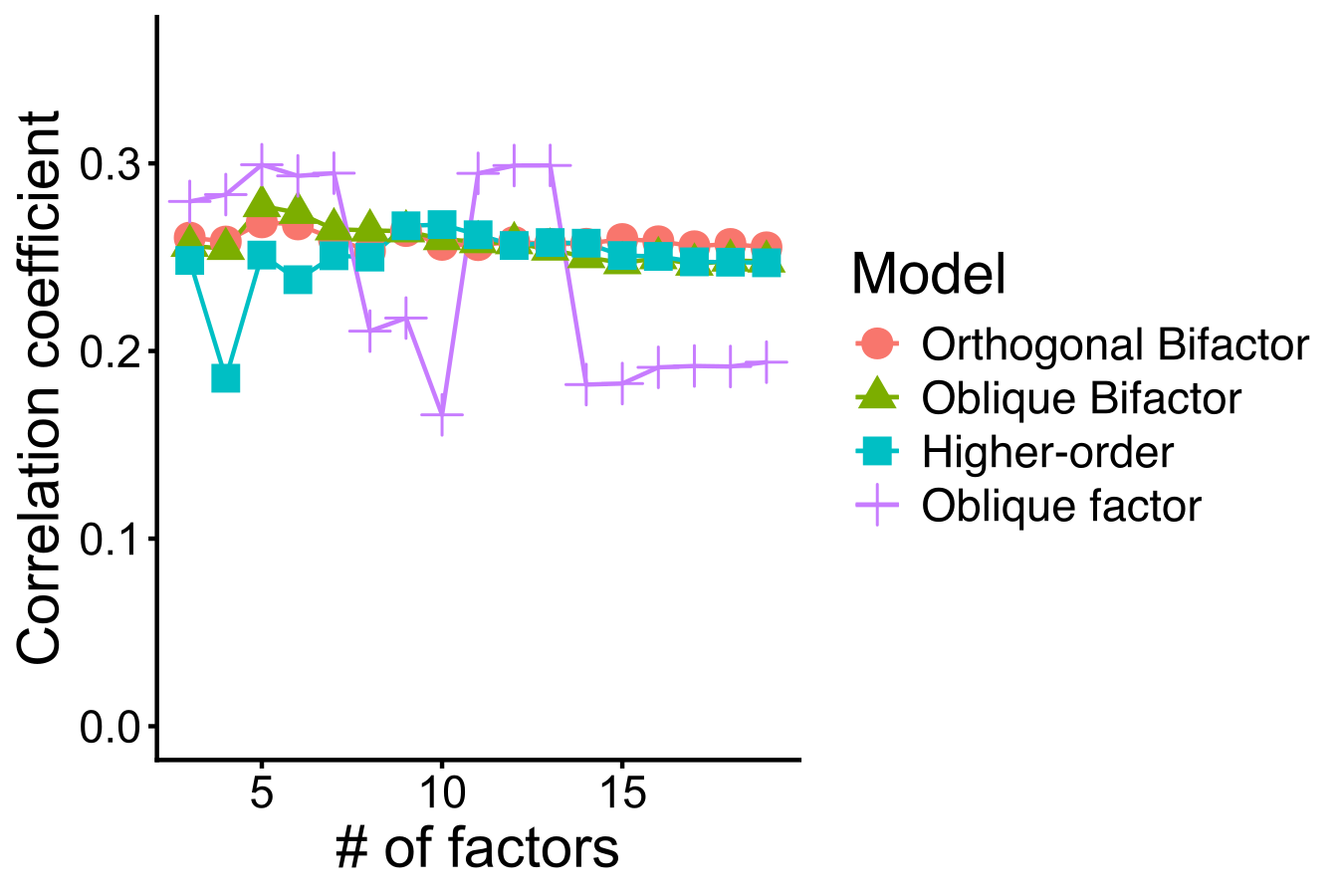


Fig O

Association strength of specific factors for diagnostic history in each factor model in Dataset-2.


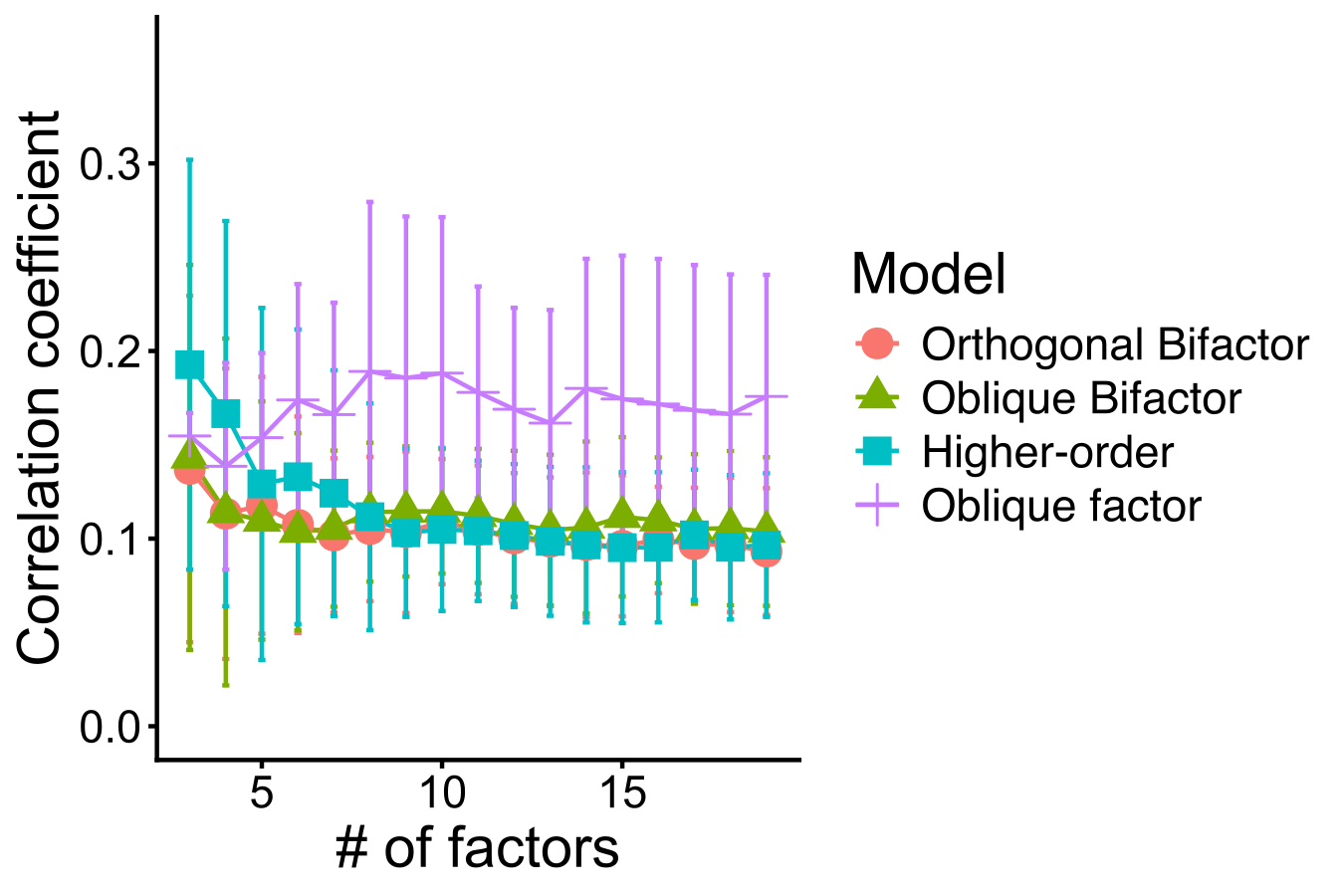


Fig P

Factor loadings of exploratory factor analysis (EFA) in Dataset-2.


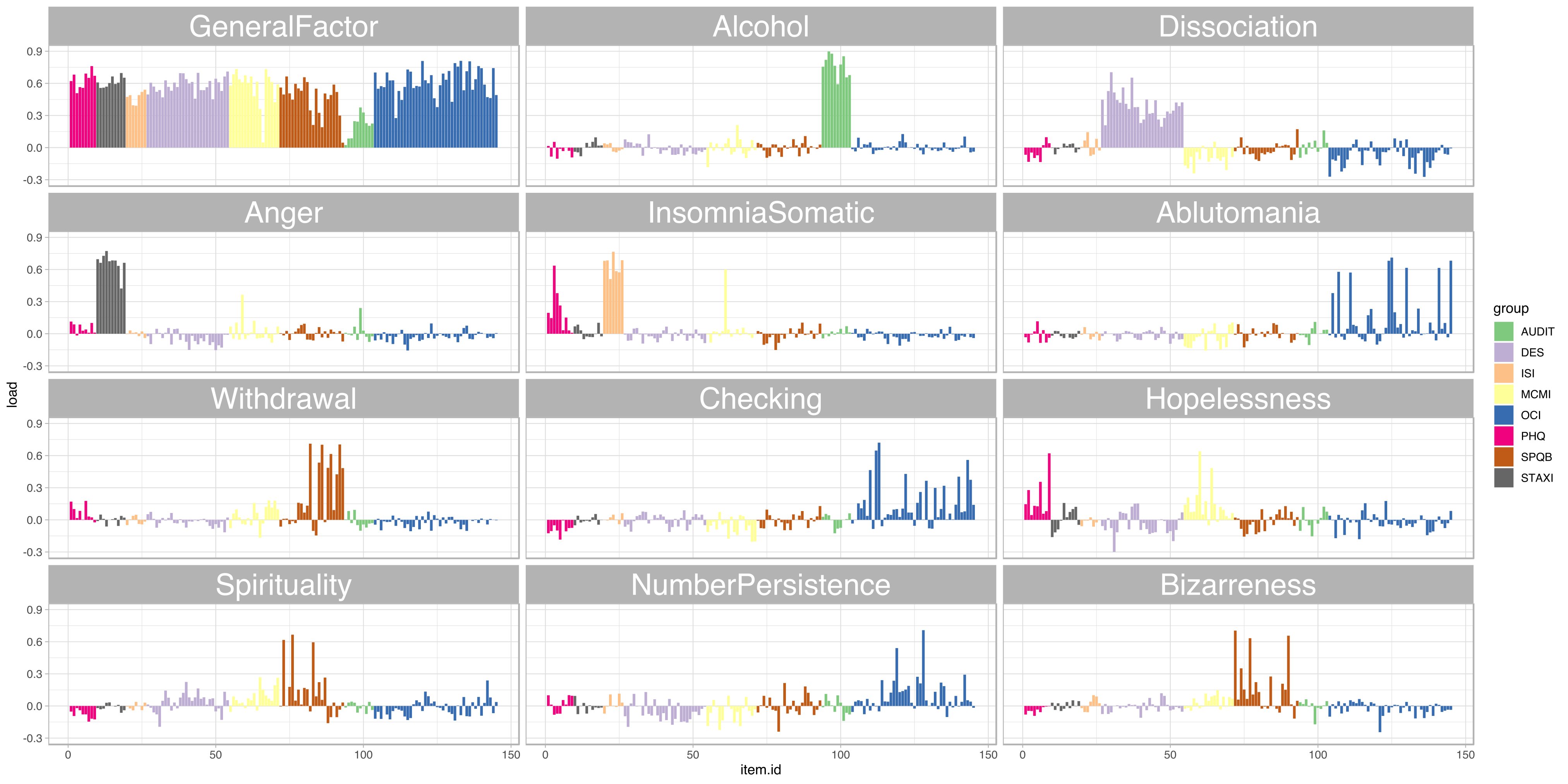


Fig Q

Factor loadings of exploratory factor analysis (EFA) in Dataset-3.


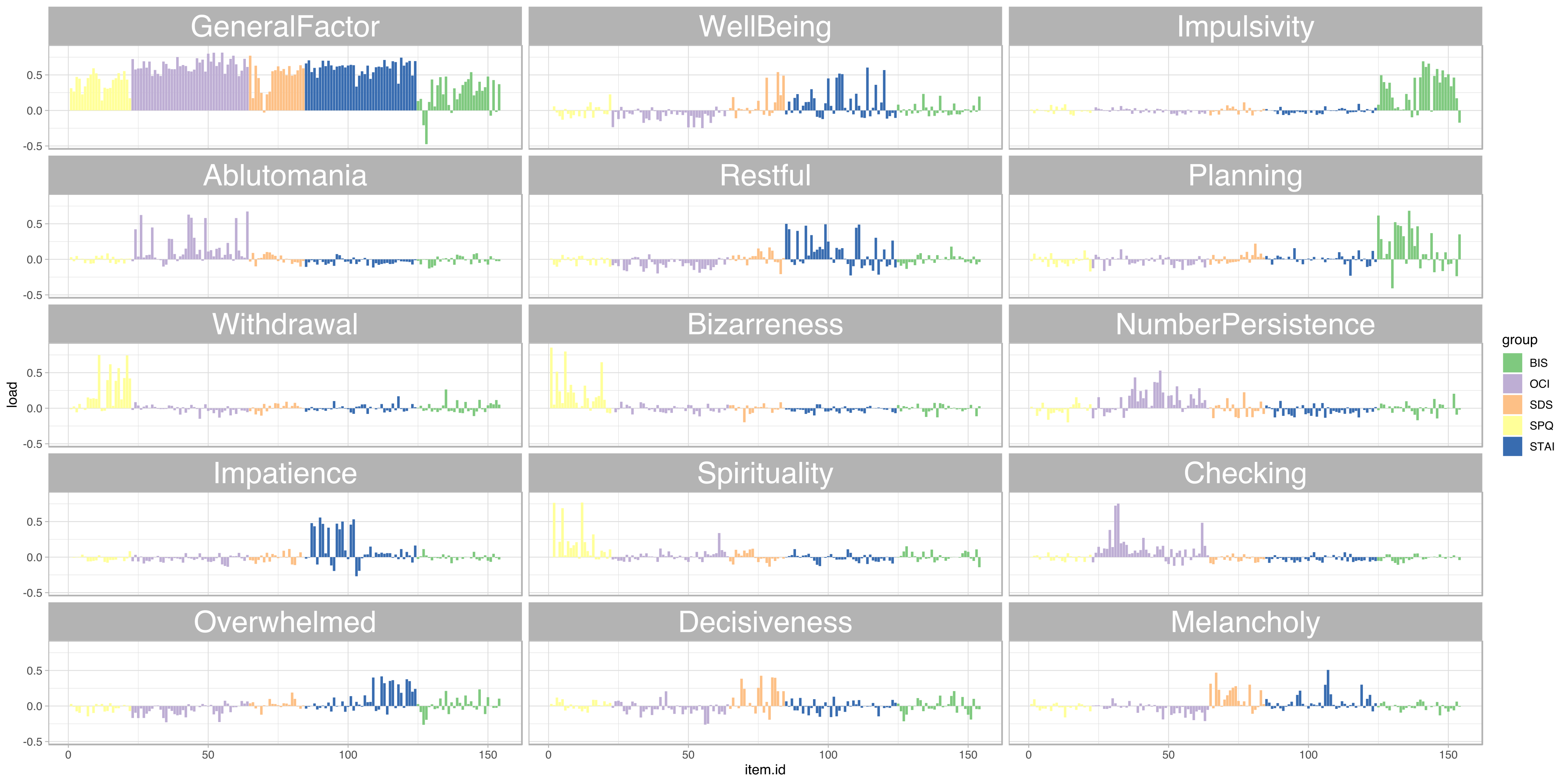


Fig R

Multiple regression analysis of general and specific factors for diagnostic history to assess convergent and discriminant validity in Dataset-2.


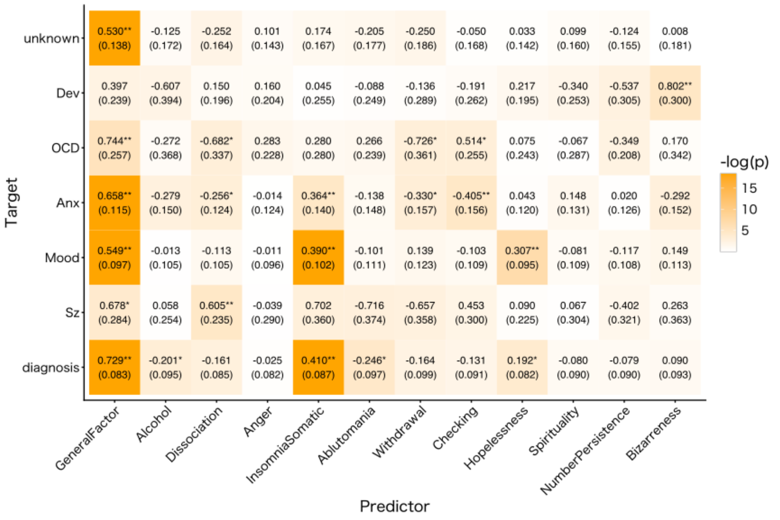


Note. Values in each cell represent regression coefficients and standard deviations. The color scale reflects the p-values, with darker colors indicating smaller p-values. Specifically, the p-values were added to a small constant (10^-8^) and then transformed into negative natural logarithms for visualization. Dev: Developmental disorders; OCD: Obsessive-compulsive disorders; Anx: Anxiety disorders; Mood: Mood disorders; Sz: Schizophrenia; diagnosis: diagnoses of any mental disorders. ** p < 0.01; * p < 0.05.

Fig S

Multi-layered hierarchy of psychiatric symptoms in Dataset-2.





Fig T

Multi-layered hierarchy of psychiatric symptoms in Dataset-3.





Fig U

Association strength of the general factors for computational phenotypes in each factor model in Dataset-3 (reward-seeking task).


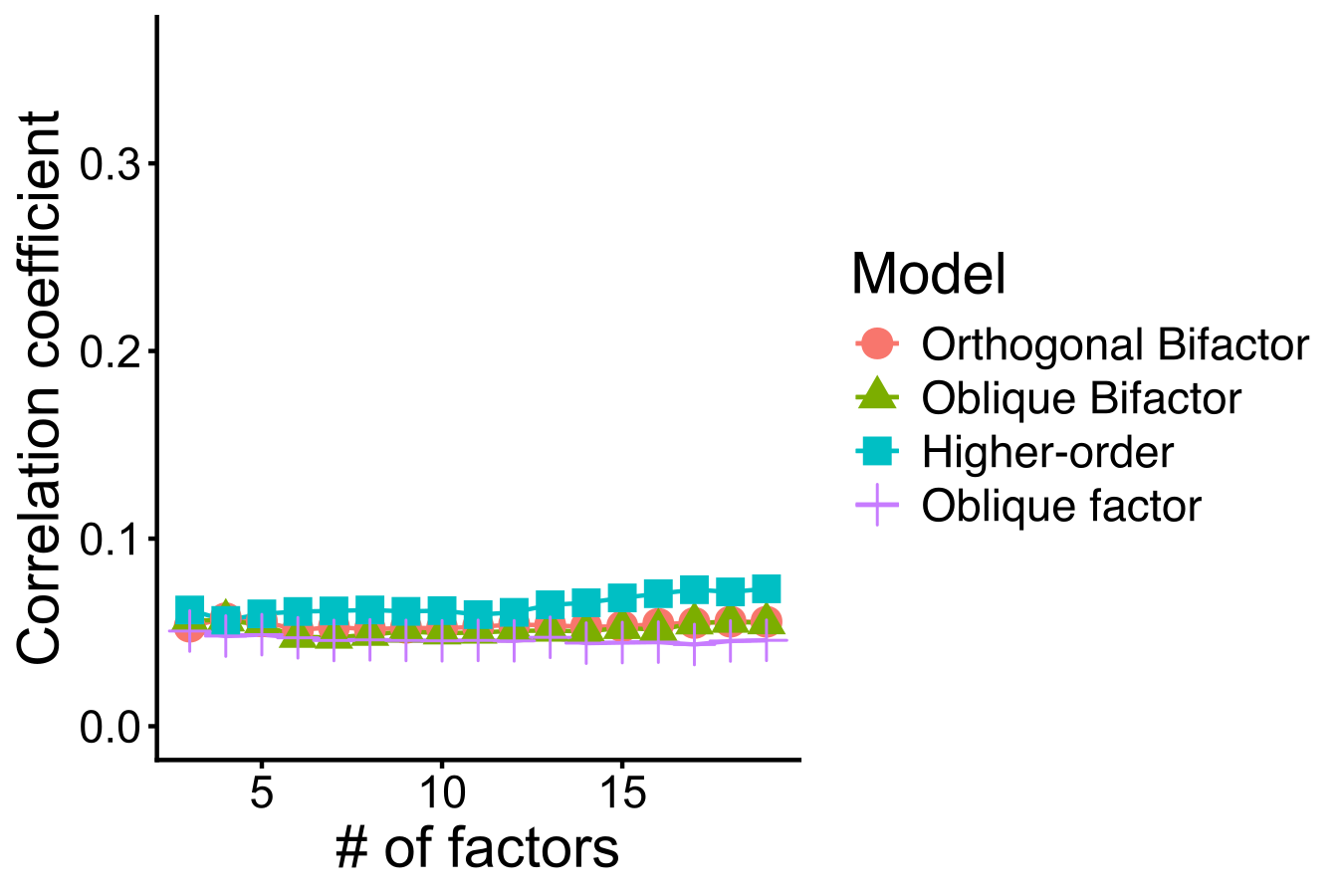


Fig V

Association strength of specific factors for computational phenotypes in each factor model in Dataset-3 (reward-seeking task).


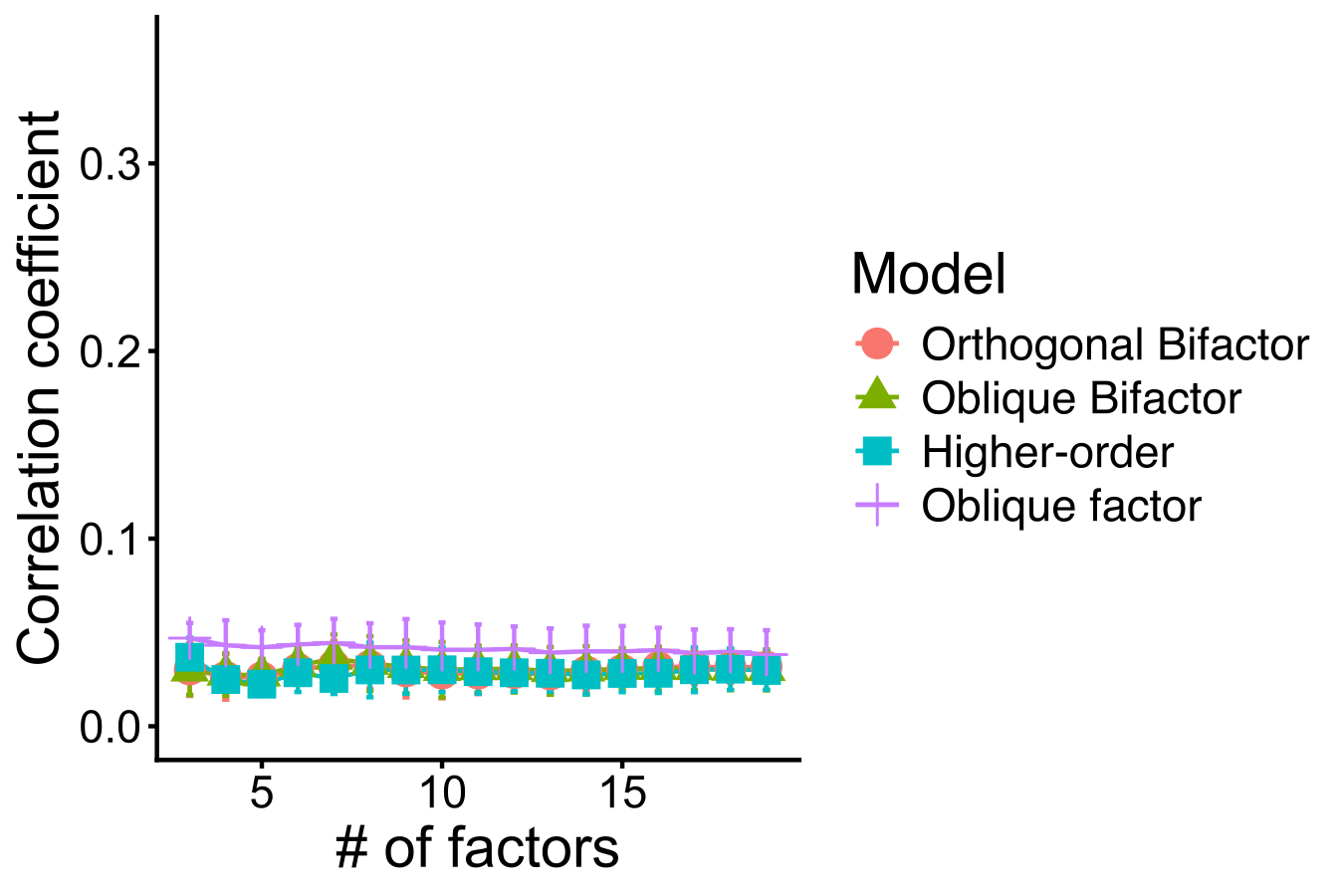


Fig W

Multiple regression analysis of the general and specific factors for computational phenotypes in Dataset-3 (reward-seeking task).


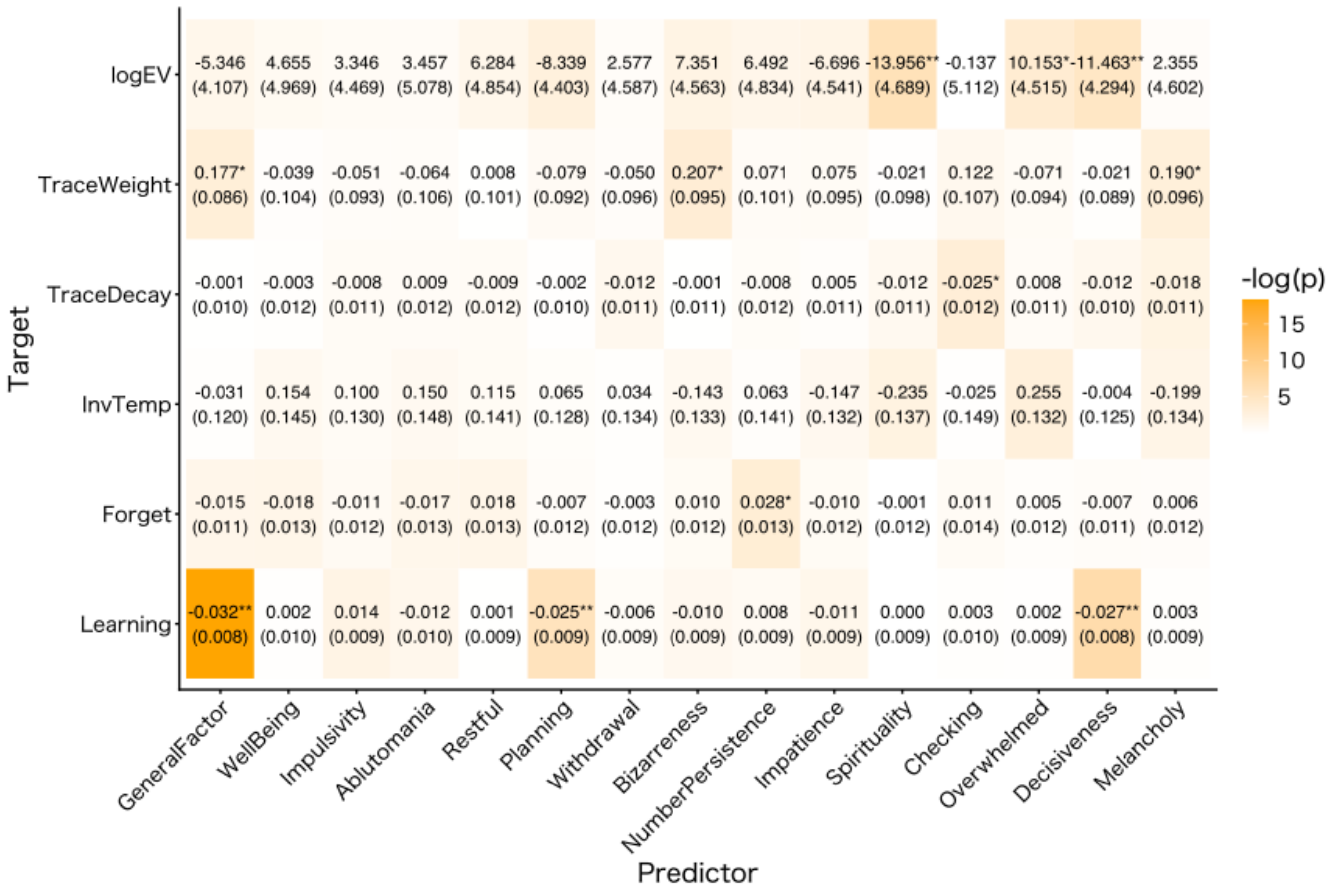


Note. Values in each cell represent regression coefficients and standard deviations. The color scale reflects the p-values, with darker colors indicating smaller p-values. Specifically, the p-values were added to a small constant (10^-8^) and then transformed into negative natural logarithms for visualization. Learning: learning rate; Forget: forgetting rate; InvTemp: inverse temperature; TraceDecay: choice-trace decay rate; TraceWeight: choice-trace weight; logEV: the logarithm of the model evidence. ** p < 0.01; * p < 0.05.

Fig X

Association strength of the general factors for computational phenotypes in each factor model in Dataset-3 (loss-avoidance task).


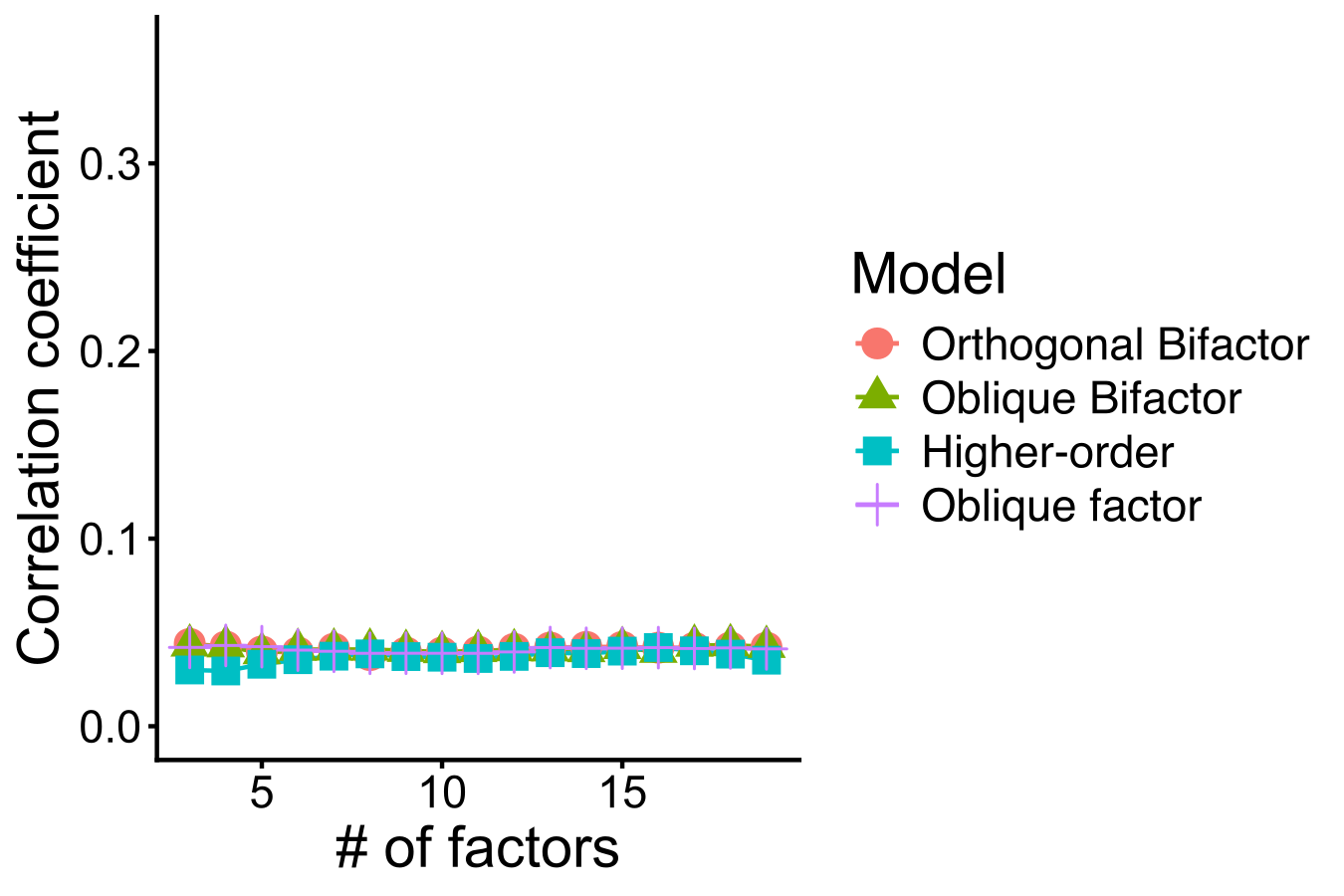


Fig Y

Association strength of specific factors for computational phenotypes in each factor model in Dataset-3 (loss-avoidance task).


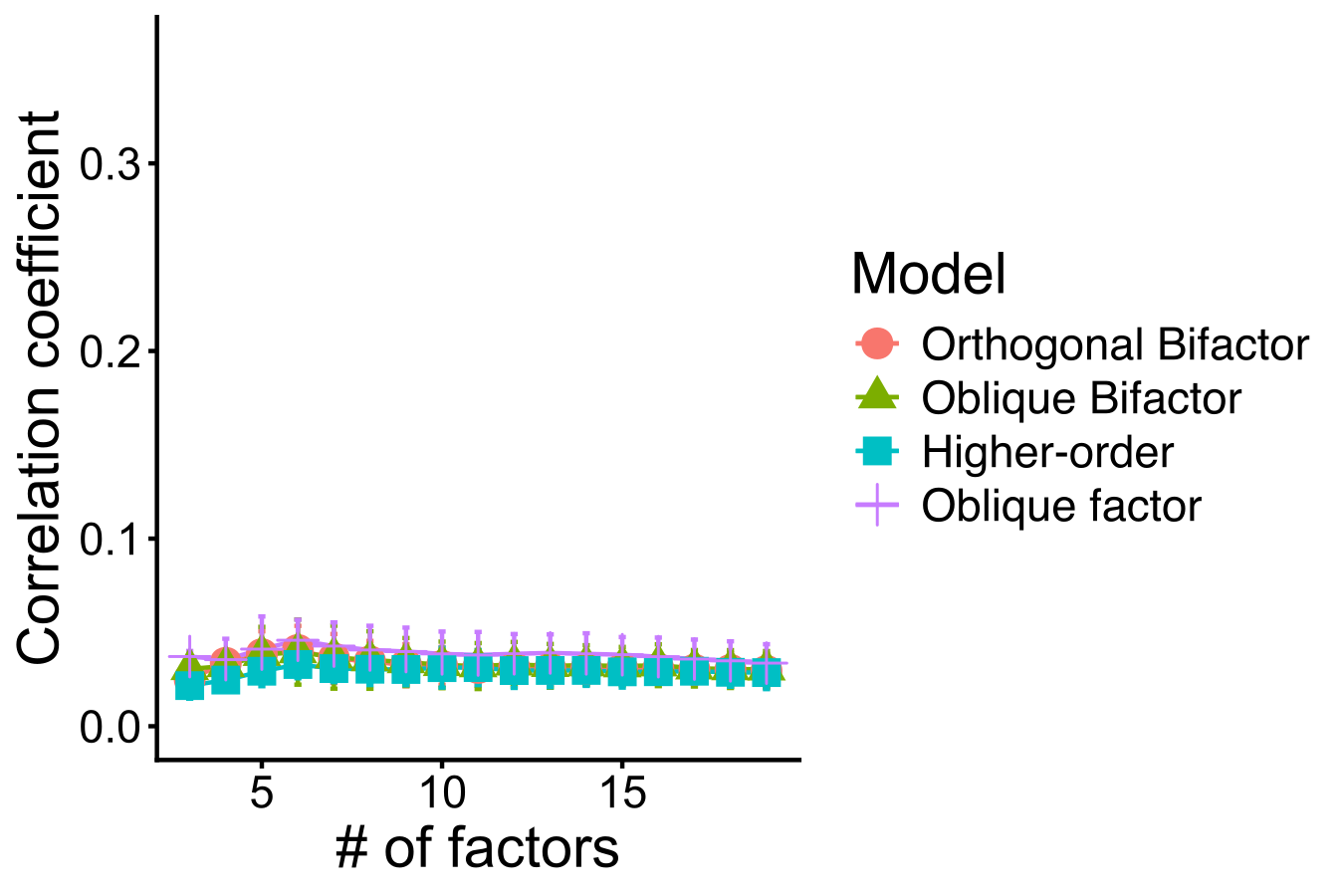


Fig Z

Multiple regression analysis of general and specific factors for computational phenotypes in Dataset-3 (loss-avoidance task).


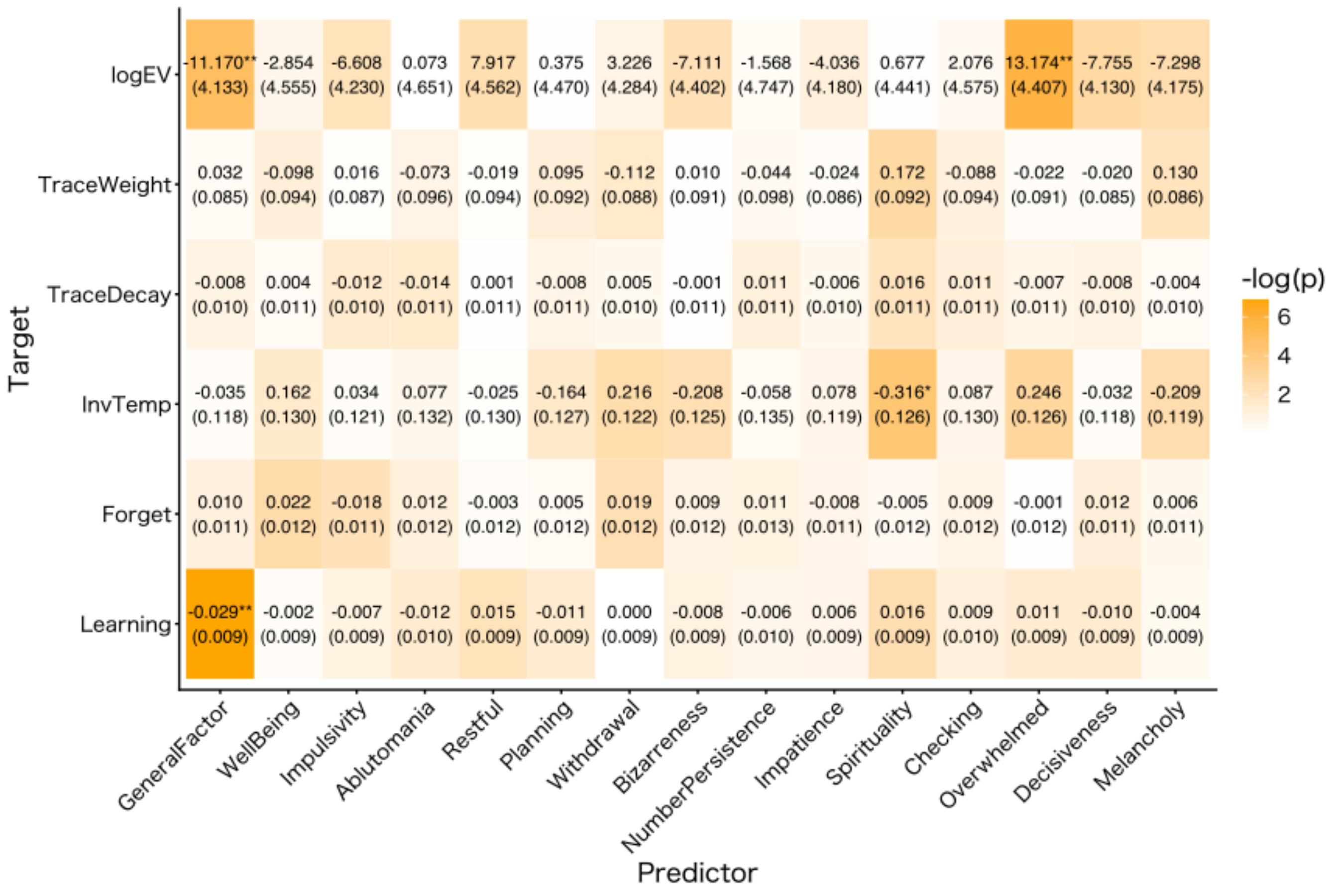


*Note*. Values in each cell represent regression coefficients and standard deviations. The color scale reflects the p-values, with darker colors indicating smaller p-values. Specifically, the p-values were added to a small constant (10^-8^) and then transformed into negative natural logarithms for visualization. Learning: learning rate; Forget: forgetting rate; InvTemp: inverse temperature; TraceDecay: choice-trace decay rate; TraceWeight: choice-trace weight; logEV: the logarithm of the model evidence. ** *p* < 0.01; * *p* < 0.05.
